# Rural/Urban and Socioeconomic Differentials in Quality of Antenatal Care in Ghana

**DOI:** 10.1371/journal.pone.0117996

**Published:** 2015-02-19

**Authors:** Patience A. Afulani

**Affiliations:** 1 Department of Community Health Sciences, Fielding School of Public Health, University of California, Los Angeles, Los Angeles, California, United States of America; 2 California Center for Population Research, Los Angeles, California, United States of America; University of Southampton, UNITED KINGDOM

## Abstract

**Background:**

Approximately 800 women die of pregnancy-related complications every day. Over half of these deaths occur in sub-Saharan Africa (SSA). Most maternal deaths can be prevented with high quality maternal health services. It is well established that use of maternal health services vary by place of residence and socioeconomic status (SES), but few studies have examined the determinants of *quality* of maternal health services in SSA. The purpose of this study is to examine the determinants of antenatal care (ANC) quality in Ghana–focusing on the role of place of residence and SES (education and wealth). The analysis also examines the interactions of these variables and the mediating role of ANC timing, frequency, facility type, and provider type.

**Methods:**

The data are from the Ghana Maternal Health Survey (N = 4,868). Analytic techniques include multilevel linear regression with mediation and moderation analysis.

**Results:**

Urban residence and higher SES are positively associated with higher ANC quality, but the urban effect is completely explained by sociodemographic factors. Specifically, about half of the urban effect is explained by education and wealth alone, with other variables accounting for the remainder. The effects of education are conditional on wealth and are strongest for poor women. Starting ANC visits early and attending the recommended four visits as well as receiving ANC from a higher level facility and from a skilled provider are associated with higher quality ANC. These factors partially explain the SES differentials.

**Implications:**

Ghanaian women experience significant disparities in quality of ANC, with poor illiterate women receiving the worst care. Targeted efforts to increase quality of ANC may significantly reduce maternal health disparities in Ghana and SSA. A particularly crucial step is to improve ANC quality in the lower level health facilities, where the most vulnerable women are more likely to seek ANC.

## Introduction

Approximately 800 women die of pregnancy-related complications every day. Ninety-nine percent of these deaths occur in developing countries, with Sub-Saharan Africa (SSA) accounting for over half of the total [1,2]. Most maternal deaths can be prevented with high quality maternal health services—antenatal, delivery, and postnatal care [3,4]. But previous efforts in SSA have concentrated more on increasing the proportion of women who *use* these services, rather than the *quality* of the services provided [5,6]. Although antenatal care (ANC) coverage levels have increased substantially, maternal mortality rates remain high. One reason is that skilled delivery care is still low; another reason is poor quality antenatal and delivery care [7–10].

The recommendations to reduce maternal mortality range from preconception to postpartum interventions. But because most maternal deaths occur during delivery, skilled attendance at delivery is critical to reducing maternal mortality [11]. Many women in SSA, however, do not use skilled attendants during delivery. The reasons include poor access to health facilities and some sociocultural factors that discourage use of health facilities for delivery. Qualitative studies also suggest that perceived poor quality of health services deters women from using skilled birth attendants, although there is little quantitative evidence to support this hypothesis [12–14].

In addition to skilled delivery care, the World Health Organization recommends that all pregnant women receive ANC at least four times in the course of their pregnancy [15,16]. However, evidence for the role of ANC in reducing maternal mortality is not consistent [17,18]. Some researchers suggest the inconsistent findings may reflect inadequate attention by researchers to the content of ANC and to problems of endogeneity [19]—i.e., the content of ANC may affect use of ANC services, and where ANC is available, women who choose to use it may be those less likely to experience maternal mortality for other reasons. While ANC alone may be insufficient to reduce maternal mortality, antenatal care visits (ANCVs) are an opportunity to reach women with interventions (e.g., blood pressure monitoring, iron supplementation, tetanus vaccination, and education about the danger signs of pregnancy), which are essential to both maternal and fetal health. ANCVs are also an opportunity to identify women with pregnancy complications and to start the appropriate management. Additionally, during ANCVs women can be educated and assisted in planning ways to access skilled care during delivery [15,20,21]. This implies that high quality ANC can help improve maternal outcomes: directly through preventative measures and early management of complications and indirectly through increased use of skilled birth attendants.

The determinants of *use* of antenatal and other maternal health services in SSA are well known. Few quantitative studies have, however, examined the determinants of quality of ANC or other maternal health care that women in SSA receive [22–24]. The paucity of studies on the quality of maternal health care is primarily due to lack of data. Quality of care is difficult to measure—it has multiple dimensions that are hard to capture in a few variables. Donabedian identified three dimensions of quality of care: (1) structure: the physical and human resources to provide care; (2) process: the technical and interpersonal aspects of delivering care; and (3) outcomes: the effects of care [25]. For maternity care, Hulton et al. regrouped elements under these dimensions into two broad categories: service provision and patient experience [26]. Service provision data may be obtained from health facility assessments, but such data are difficult, if not impossible, to link to individual women; and patient experience data necessarily requires that we ask individual women. These factors coupled with the weakness of routine systems for collecting health information means that we have to rely on household surveys in which women are respondents. The nature of surveys, however, limits the detail and reliability of data on quality of care because women may not be able to assess and report on the technical aspects [6]. Nonetheless, household surveys provide the best population-level source of information on the quality of ANC women receive; but few researchers have taken advantage of them to examine social disparities. This paper uses data on antenatal service provision in the Ghana Maternal Health Survey (a special supplement to the Ghana Demographic and Health Survey (DHS)) to examine factors associated with the quality of ANC that women in Ghana receive.

More than 95 percent of women in Ghana go for at least one ANCV during pregnancy, and about 80 percent attend the recommended four or more ANCVs. But only about two thirds of women deliver with a skilled birth attendant, with wide disparities by place of residence and socioeconomic status (SES). For instance, only about 24 percent of the poorest women deliver with a skilled birth attendant, compared to over 95 percent of the richest women [27,28]. The maternal mortality for Ghana also remains high, at 380 per 100,000 live births, despite efforts by the Ministry of Health and other organizations to reduce it [2]. Like many countries in SSA, Ghana is not on track to achieve the Millennium Development Goal number 5—to reduce the maternal mortality ratio by three quarters between 1990 and 2015 [2,29]. The low use (as well as late use) of skilled birth attendants is contributing to the high maternal mortality in Ghana. The other reason is poor quality of delivery services in some health facilities [30]. Little is known about the quality of ANC that Ghanaian women receive or the factors associated it. But studies elsewhere have found rural/urban and SES disparities in ANC quality, though none has examined the factors underlying the disparities [19,22–24]. Disparities in the quality ANC may therefore be contributing to the disparities in the use of skilled birth attendants and potentially to the high maternal mortality in Ghana.

This study has two aims. The first aim is to identify the factors associated with the quality of ANC that Ghanaian women receive. The second aim is to examine how social factors—urban/rural residence and SES (education and wealth)—affect quality of ANC received. For this second aim, I examine the interactions between the social factors, and the factors potentially explaining the social differentials. Drawing on existing literature, I hypothesize that higher SES and urban residence will be associated with higher quality ANC. But I expect that the lower SES of rural women will partly explain the rural/urban difference in quality of ANC. I also examine the reverse of this hypothesis—whether place of residence explains the SES difference in quality of ANC. If SES explains most of the rural/urban difference, it suggests that higher quality services are more accessible to the higher SES women. On the other hand, if place of residence explains most of the SES difference, it suggests general poor quality of care in rural areas, which is not greatly affected by a woman’s SES. I also examine conditional effects: whether the effect of education and wealth differ by place of residence, and whether the effect of education differs by wealth. This analysis will help identify women who are least likely to receive high quality ANC. Finally, I examine the timing and frequency of ANCVs and the type of ANC facility and provider as potential mediating factors for the rural/urban and SES effects. This part of the analysis will assess whether the disparities in quality of ANC by place of residence and SES are due to differences in the use of antenatal services or to use of different types of facilities and providers.

## Methods

### Data

The data for this analysis are from the 2007 Ghana Maternal Health Survey (GMHS) [31]. The GMHS was the first (and still is the only) nationally representative population-based survey to collect comprehensive information on maternal morbidity and mortality in Ghana. The survey was conducted by the Ghana Statistical Service and the Ghana Health Service, with technical assistance from Macro International. The main aim of the GMHS was to generate data on maternal health and mortality for policymakers and the research community involved in the Reducing Maternal Morbidity and Mortality Program. Data collection was implemented in two phases. First, a short household questionnaire was administered in 227,715 households, which were randomly selected from 1600 primary sampling units in urban and rural areas in the ten administrative regions of the country. The goal was to identify female deaths between ages 12–49 years. In the second phase, 400 clusters were randomly selected from the 1600 clusters included in phase I. Households with women age 15–49 years were selected at random from these 400 clusters, stratified by region and rural/urban residence. Institutional populations and those residing in refugee camps were excluded. Verbal autopsies were completed for maternal deaths in the selected households. A household questionnaire and a women’s questionnaire was also administered in the selected households to collect information on demographic and health indicators. This yielded 10,858 completed household interviews and 10,370 individual interviews with women aged 15–49 years. Face-to-face interviews were conducted in English and three major local languages: Akan, Ga, and Ewe. The response rate was 99% at the household level and 98% for the individual women. The GMHS is described in detail in the published survey report [31].

The questions on antenatal care were asked of only women who had a birth (live or still birth) in the five years preceding the survey (N = 5,088 = 49.1% of all women interviewed); this is the base sample for this analysis. The analytic sample is however 5,042 women (99.1% of the base sample) because 46 observations are missing on key study variables. The analysis is further restricted to women who had at least one ANCV during their last pregnancy, since quality of ANC cannot be measured for women who did not have an ANCV; 97% (N = 4,868) of women in analytic sample had at least one ANCV.

### Ethics statement

This study was granted an exemption under the University of California, Los Angeles Institutional Review Board exemption category 4 for research involving the study of existing data.

### Constructs and variables

Dependent variable: Quality of antenatal care

In this analysis, quality of ANC is defined as receipt of the recommended ANC services during pregnancy. This definition is based on the definition of quality of care proposed by Donabedian: the extent to which actual care is in conformity with present criteria for good care [32]. I created an additive index of responses to nine questions on ANC services that women received during their last pregnancy. The services are: being weighed, blood pressure checked, a urine sample taken, a blood sample taken, education received on signs of pregnancy complications, education received on where to go in the event of a complication, received or told to buy iron supplements, received an anthelminthic, and received tetanus vaccination. Each question has a binary response (1 = Yes; and 0 = No). Women were also asked if they had received a tetanus vaccination at any time before pregnancy, and how many times. Four tetanus injections are required for full protection [33]. Thus, women who reported receiving at least four injections prior to the index pregnancy were coded as having received a tetanus injection, even if they did not receive it during the index pregnancy. An exploratory principal components analysis (PCA) of these variables yielded one dominant factor. I, however, decided to use the additive index because of the problems of PCA with binary variables [34] and because the sum is easier to interpret. Observations missing on one or more of the component variables were assumed to be zero; no observations were missing on all the component variables. This approach may underestimate the quality of care because women who did not know whether they received a service are counted as having not received it. However, the number of cases included for this reason is very small. The index ranges from zero to nine with responses spanning the entire range; the mean is 7.4.

Key independent variables: Place of residence and socioeconomic status

Place of residence refers to whether the respondent lives in a rural or urban area. Urban areas are defined as localities with 5000 or more persons, while rural areas are localities with less than 5000 persons [35]. Place of residence captures the general quality of services in the area where each respondent lives, as well as other factors including the accessibility of health services. The other variable related to place of residence is the region where one lives. I control for region to capture unmeasured contextual factors. There are ten administrative regions in Ghana, with Greater Accra region being home to Accra—the national capital of Ghana.

SES refers to the social rank of an individual and her family in a particular community or society. It incorporates economic status usually measured by income and/or wealth and social status usually measured by education and/or occupation [36]. While some researchers use a composite measure of SES, I use separate measures, because each may have a different effect [37]. Including the individual components of SES also makes it easier to identify plausible explanatory pathways and mechanisms through which SES affects health outcomes [37]. I operationalize SES in this analysis as education and wealth. I examine education both as a categorical variable (highest level of education attained by respondent) and as continuous variable (years of education attained), but I use years of education in the final models because the two specifications produce similar results. Years of education is top coded at 12 years of education, as there are very few women with more than 12 years of education in the sample; and centered at the sample mean to reduce collinearity with the interaction terms and to aid interpretation [38,39]. Wealth is measured in quintiles. The quintiles were calculated from a wealth index based on principal component analysis of variables on household assets conducted by the group that produced the data files [40].

Potential mediating variables: Antenatal care timing, frequency, facility, and provider

There are other factors that may affect the quality of ANC that a woman receives independent of where she lives and her SES. These include the timing of the first ANCV, the number of ANCVs, the type of ANC facility, and the type of ANC provider. Women who start ANC in the first trimester and attend the recommended four or more times are more likely to receive all the essential ANC services, because contact with the health system begins earlier and is more frequent. Higher-tiered facilities like hospitals may also provide better quality ANC than lower-tiered ones like health centers, because of better infrastructure and personnel; and private health facilities may provide better quality care than public health facilities, although this is more likely for interpersonal than technical quality [41,42]. In addition, the quality of care received may differ depending on whether the provider is a doctor, nurse/midwife, or other provider, because of different skill levels and access to amenities (e.g., laboratory services) [43]. Prior studies and preliminary analysis for this study show that place of residence and SES are associated with both quality of ANC and ANC timing, frequency, type of facility, and type of provider [19,22,23]. Thus, these factors may account for some of the effects of both place of residence and SES on quality of ANC. Prior studies have combined timing and frequency of ANCVs with the content of the ANCVs (the services received) to measure quality of ANC and adequacy of ANC. Examining these factors separately is however important in assessing the underlying causes of disparities.

For the analysis, the number of ANCVs is dichotomized to ‘one to three’ and ‘four or more visits,’ based on the number of ANCVs recommended by the World Health Organization [16]. The trimester of the first ANCV is coded as first, second, and third trimester, and don’t know. The categories for the type of ANC facility are: government hospital or polyclinic; government health center, health post, or other lower tiered government health facility; private facility (including maternity homes); and ‘other,’ representing ANC outside a facility, including the home of the provider or the woman. The categories for type of ANC provider are: doctor; nurse or midwife; and ‘other,’ representing unskilled providers (auxiliary nurse or midwife, traditional birth attendant, or any provider other than a doctor, nurse or midwife). If a respondent received ANC in more than one type of facility or from more than one type of provider, the highest level facility and most highly trained provider are used.

Other independent variables

Other factors that may influence the quality of care received include women’s risk for adverse pregnancy outcomes. For instance, women who had prior pregnancy complications or complications in the index pregnancy may receive higher quality care because health workers may pay greater attention to them[13]. Women who know they are at higher risk for adverse outcomes may also actively seek better care. In addition, a woman’s age, gravidity (number of pregnancies), or parity (number of births) may influence the type of care she receives. I therefore control for age, gravidity, parity, having a prior stillbirth or miscarriage, having a sister who died from maternal causes, experiencing any complication during the pregnancy (i.e. reported signs or symptoms of hemorrhage, preeclampsia, eclampsia, infection, obstructed labor, etc.), experiencing a serious complication during the pregnancy (i.e., a problem for which she sought care), and the reason for antenatal care (whether for checkup or for a problem). Familiarity with the health system may also influence the quality of care received because women learn where to seek high quality care. I include two variables—ever used contraception and knowledge of where to get contraception—as proxies for familiarity with the health system. In addition, women’s status and autonomy may enable them to advocate within their families and at health facilities to receive better quality services. SES captures autonomy and status, at least in part. But I also control for marital status, age at first union, and sex of the household head (female headed household or not) as other proxies for women’s autonomy [13]. Finally, I control for religion and ethnicity to capture sociocultural factors that may influence the quality of care that a woman receives.

### Analytic approach

Initial analyses involved descriptive statistics for the sample—means for continuous variables and proportions for categorical variables. Next I examined the bivariate associations between the independent variables and the dependent variable. The descriptive statistics and cross tabulations are all weighted using the sample weights provided with the data to account for the complex sampling design. I used t-tests to assess statistical significance for the mean differences in quality of care for binary independent variables; analysis of variance (ANOVA) for non-binary categorical independent variables; and correlations for continuous variables. Chi-squared tests are used to examine for differences by place of residence, educational level, and wealth [44–46]. To account for the hierarchical nature of the data, I estimated bivariate and multivariate multilevel linear regression models. The use of multilevel regression is necessary because clustering at various levels or groups results in underestimation of the standard errors, such that, when clustering is not accounted for there is a higher chance of finding significant differences, when in fact the differences are not significant [47,48]. The levels included in this analysis are individual (level 1), cluster (level 2), and district (level 3). The weights provided with the datasets are only for the individual level, and there is not enough information to construct weights for use in the multilevel analysis. Furthermore, there is no clear consensus on the use of weights in multilevel analysis within the field of statistics [49]. I therefore estimated unweighted multilevel models. The “xtmixed” command in Stata was used to estimate the multilevel linear regression models with random intercepts [48,50].

I estimated a multilevel linear regression model for quality of ANC to identify the factors associated with quality of ANC—controlling for other factors and inter-district and inter-cluster variation. To determine whether the effects of place of residence, education and wealth are conditional on each other, I examined three interactions: place of residence and education, place of residence and wealth, and education and wealth. I show only statistically significant interactions, in this case only the education and wealth interaction. Mediation models, using the difference of coefficients (c-c’) method, were used to determine whether SES accounts for some of the rural/urban difference (and the reverse), and whether the effects of both are through ANC frequency, timing, facility, and provider (approach of the mediation analysis is illustrated in S1 Fig.) [38,39,51].

## Results

### Descriptive results

Table 1 shows the weighted and unweighted distributions of the key variables (distributions for full set of variables in S1 Table). About two-thirds of the women live in rural areas. All regions of Ghana are represented in the sample, contributing between 8% and 13% except for the Ashanti region (the most populous region), which makes up 19%, and Upper East and Upper West regions (the least populous regions), which make up only 5% and 3% respectively. The average number of years of education is about 5, with a range of 0 to 18 years. About a third of the women have no formal education; 22% have only a primary education; 37% have middle or junior secondary school (JSS) education; and less than one in ten women (7%) have completed secondary or senior secondary school (SSS) (Middle and JSS are equivalent and secondary and SSS are also equivalent—the names changed with changes in the educational system). The household wealth index places women into five quintiles based on household assets; thus, there are about 20% in each group. About three-quarters of the women identified as Christian; 18% identified as Moslem and 9% as traditionalist, spiritualist, other, or have no religion. Akans, the dominant ethnic group in Ghana, make up close to half of the sample (47%), with Ewes at 12%, and Gas, Dangmes, and Guans at 9%.

**Table 1:** Distribution of key study variables, Women who attended ANC at least once, GMHS, N = 4,868.

	*Unweighted*		*Weighted*		
*Variables*	N	%	proportion	[95% C.I]	
**Setting**					
Rural	2,967	61.0	0.648	0.617	0.679
Urban	1,901	39.1	0.352	0.321	0.383
**Highest Education**					
None	1,588	32.6	0.330	0.296	0.364
Primary	1,072	22.0	0.221	0.202	0.239
Middle/JSS	1,804	37.1	0.375	0.345	0.404
Secondary/SSS/or higher	404	8.3	0.075	0.063	0.086
Mean years education (SD)	4,868	5.2 (4.39)	5.130	4.815	5.444
**Household wealth index**					
Poorest	1,024	21.0	0.207	0.177	0.236
Poorer	943	19.4	0.210	0.186	0.235
Middle	930	19.1	0.204	0.182	0.227
Richer	976	20.1	0.203	0.181	0.224
Richest	995	20.4	0.176	0.155	0.197
*ANC variables*					
**ANC quality of care score**					
Mean (SD)	4,868	7.4 (1.52)	7.406	7.322	7.490
**No. of ANC visits**					
1–3 visits	990	20.3	0.202	0.184	0.221
Four or more	3,878	79.7	0.798	0.779	0.816
Mean (SD)	4,868	5.8 (2.75)	5.756	5.626	5.885
**Trimester of first ANC visit**					
First trimester	2,688	55.2	0.549	0.529	0.568
Second trimester	1,992	40.9	0.413	0.396	0.431
Third trimester	181	3.7	0.036	0.030	0.042
Don't know	7	0.1	0.002	0.000	0.003
**Where ANC took place**					
Gov't health facility only/combine	4,119	84.6	0.853	0.829	0.877
Gov't hospital or polyclinic	2,200	45.2	0.453	0.413	0.492
Other Gov't facility	1,919	39.4	0.400	0.361	0.439
Only Private facility/maternity home	703	14.4	0.140	0.116	0.164
Home/other/DK	46	0.9	0.007	0.005	0.010
**Highest trained ANC provider**					
Doctor	1,006	20.7	0.194	0.176	0.213
Nurse	3,743	76.9	0.785	0.766	0.803
All others	119	2.4	0.021	0.015	0.026
**Reason for seeking ANC**					
For checkup	4,044	83.1	0.831	0.817	0.846
For a problem/9missing	824	16.9	0.169	0.154	0.183
**Mean Age in years (SD)**	4,868	30.5 (7.34)	30.426	30.183	30.670
**Marital status**					
Currently married	3,510	72.1	0.718	0.697	0.739
Cohabiting	666	13.7	0.141	0.125	0.157
Previously married	347	7.1	0.070	0.061	0.078
Never married	345	7.1	0.071	0.062	0.080

Notes: ANC = Antenatal Care; GMHS = Ghana Maternal Health Survey (2007); JSS = Junior Secondary School; SSS = Senior Secondary School; CI = Confidence interval; SD = standard deviation; DK = don’t know.

The average age is about 31 years; age ranges from 15 to 49 years—the target group for the survey. Most women are currently married (72%); 14% are cohabiting. On average, the women have between three and four children. Twenty percent had a prior adverse pregnancy outcome (stillbirth or miscarriage); 20% had some pregnancy complication during their last pregnancy; and 17% had a complication for which they sought help. Two percent had a sibling who died from maternal causes. About two-thirds (62%) have ever used some type of contraception, and about half (53%) know where to get contraception. The sample distribution is similar for the full sample (i.e., including the 3% (N = 174) of women who did not receive any ANC during pregnancy).

The average number of services received by women who attended at least one ANCV is 7.4 (95% CI: 7.32 to 7.49), with a range of zero to nine; 10% received five or fewer services, and about a quarter received all nine services. About 80% had four or more ANCVs, with an average of about six visits; and 55% started ANCVs in the first trimester. Eighty-five percent received ANC in a government health facility, with roughly half in a hospital or polyclinic and the other half in a health center or other lower tiered facility; 14% received ANC in a private facility. Most women (79%) received ANC from a nurse or midwife; 19% received some ANC from a doctor; and less than 3% received ANC from an unskilled provider. Most women went for the ANCV for a checkup; 17% went because of a problem.

### Weighted bivariate results

The bivariate statistics for the key study variables are shown in Table 2 (full set of bivariate statistics in S2 Table). The results show small but significant differences in ANC quality by place of residence, education, and wealth. On average, women who live in urban areas receive higher ANC quality than those in rural areas (7.7 vs. 7.2). ANC quality increases with education and wealth: the average ANC quality score among women with no education is 7.0, compared to 7.7 for those with middle/JSS or higher; and 6.9 among the poorest compared to 7.8 among the richest. The ANC quality also differs significantly by the timing and frequency of ANCVs as well as by the type of ANC facility and provider. The average quality score among women who had less than four ANCVs is 6.6, compared to 7.6 for those who attended four or more times; and 6.3 for women who started ANCVs in the third trimester, compared to 7.6 for those who started in the first trimester. Women who received ANC in a government hospital or polyclinic received higher quality ANC than those who did so in other facilities; but there is no difference in ANC quality between women who received ANC in private facilities and those who received it in lower level government health facilities. Also, women who received ANC from a doctor had a small but significantly higher ANC quality than those who received ANC from a nurse or midwife, which is also higher than that from a provider other than a doctor, nurse, or midwife. There is no significant difference in ANC quality by reason for seeking care.

**Table 2:** Cross tabulation of selected variables by mean quality of antenatal care, GMHS, N = 4,868.

*Variable*	N	Mean	[95% C.1]	
**Setting**				
Rural	2,967	7.24	7.13	7.36
Urban	1,901	7.71	7.62	7.79
**Highest Education**				
None	1,588	7.03	6.88	7.17
Primary	1,072	7.36	7.24	7.47
Middle/JSS	1,804	7.71	7.61	7.80
Secondary/SSS/higher	404	7.74	7.58	7.89
**Household wealth index**				
Poorest	1,024	6.85	6.65	7.05
Poorer	943	7.26	7.12	7.39
Middle	930	7.53	7.41	7.65
Richer	976	7.63	7.50	7.75
Richest	995	7.84	7.75	7.93
**No. of ANC visits**				
1–3 visits	990	6.60	6.41	6.79
Four or more	3,878	7.61	7.54	7.68
**Trimester of first ANC visit**				
First trimester	2,688	7.59	7.51	7.68
Second trimester	1,992	7.26	7.16	7.36
Third trimester	181	6.31	5.97	6.66
Don't know	7	5.97	4.92	7.02
**Where ANC took place**				
Gov't hospital or polyclinic	2,200	7.73	7.65	7.81
Other Gov't facility	1,919	7.12	6.96	7.27
Only Private facility/maternity home	703	7.33	7.16	7.49
Home/other/DK	46	4.91	3.96	5.85
**Highest trained ANC provider**				
Doctor	1,006	7.73	7.63	7.83
Nurse	3,743	7.35	7.25	7.44
All others	119	6.59	6.10	7.08
**Reason for seeking ANC**				
For checkup	4,044	7.37	7.28	7.46
For a problem/9missing	824	7.57	7.45	7.70
**Marital status**				
Currently married	3,510	7.42	7.34	7.51
Cohabitating	666	7.18	6.95	7.41
Previously married	347	7.52	7.27	7.77
Never married	345	7.58	7.39	7.76
**Ever used contraception**				
No	1,780	7.01	6.88	7.15
Yes	3,088	7.64	7.57	7.71
**Know family planning source**				
No	2,270	7.27	7.16	7.38
Yes	2,598	7.52	7.43	7.62

Notes: See Table 1 for abbreviations.

The Western region has the highest ANC quality (8.3), and the Northern and Volta regions have the lowest (6.7). There are no major differences between the other regions except for the Greater Accra region, which surprisingly has the third lowest ANC quality—only higher than Northern and Volta regions. By religion, women who identify as traditionalists, spiritualists, other, or have no religion received the lowest quality ANC, followed by Moslems, but the quality score for Moslems is not significantly different from that of Christians. There are no significant differences in ANC quality by age, marital status, age at first union, sex of household-head, gravidity, parity, prior miscarriage or stillbirth, sibling maternal death, and any pregnancy complication. But women who have ever used contraception, women who know where to get contraception, and women who experienced a serious pregnancy complication received slightly higher quality of care than their respective reference groups.

Other bivariate analyses show very little variation in the proportion of women who had at least one ANCV by place of residence, sociodemographic, and reproductive health variables, with over 90% in all categories. More educated and wealthier women are less likely to live in rural areas than less educated and poorer women. More educated, wealthier, and urban women are also more likely to start ANCVs earlier, go for at least four ANCVs, receive ANC in a government hospital or polyclinic or a private facility, and less likely to receive ANC in a lower level facility. They are also more likely to receive ANC from doctors (S3 Table).

### Multilevel linear regression results

Table 3 shows the variances from the random effects part of the multilevel analysis of quality of ANC for the null, full unconditional, and full conditional models. The results from the null model show that, though more than half (58%) of the variation in quality of care is at the individual level, there is large variation at the district and cluster levels—Intraclass Correlations (ICC) of 0.27 and 0.15 respectively. The large ICC at the district and cluster levels supports the need for multilevel analysis [39,47]. The variances from the full models show that the independent variables in the model explain much more of the group level variation than the individual level variation; over half of the district level variation are explained by the variables in the model.

**Table 3:** Random effects from multilevel linear regression of quality of antenatal care on relevant independent variables, GMHS, N = 4,868.

*Level*	*Quality of ANC*: *variance (se)*			ICC	No. of groups	Mean observations per group
	Null model	Full unconditional model	Full conditional model			
District—level 3	0.626***	0.276***	0.275***	0.268	110	44.3
	(0.056)	(0.039)	(0.039)			
Cluster—level 2	0.352***	0.238***	0.239***	0.15	400	12.2
	(0.0336)	(0.036)	(0.036)			
Individual—level 1	1.361***	1.305***	1.305***			
	(0.014)	(0.014)	(0.014)			
N	4,868	4,868	4,868			

Notes: *p<0.05, ** p<0.01, *** p<0.001. Standard errors in parentheses.

ICC = Intraclass Correlation, calculated from the variances from the null model.

Variation at the level of the individual = 1.361/(1.361+0.626+0.352) = 1.361/2.339 = 0.582.

ICC at district level = 0.626/2.339 = 0.268; and at cluster level = 0.352/2.339 = 0.15.

The second column of Table 4 shows the multilevel linear regression results from the unadjusted models. After accounting for inter-cluster and inter-district variation, the individual factors positively associated with higher quality ANC are: living in an urban area, higher education, higher wealth, attending four or more ANCVs, going for the first ANCV in the first trimester, receiving ANC from a doctor, receiving ANC in a government hospital or polyclinic, experiencing a serious pregnancy complication, ever used contraception, knowledge of where to get contraception, being Akan, and living in the Western region.

**Table 4:** Multilevel linear regression of ANC quality on relevant independent variables, GMHS, N = 4,868.

	*Quality of ANC*: *b (se)*		
	Bivariate	Full Multivariate models	
*Independent variables*		Unconditional	Conditional
**Urban residence**	0.36***	0.084	0.084
	(0.068)	(0.066)	(0.066)
**Years of sch. centered**	0.040***	0.018**	0.043**
	(0.0054)	(0.0059)	(0.013)
**Wealth** (ref = poorest)			
Poorer/Middle	0.26***	0.17**	0.073
	(0.062)	(0.060)	(0.074)
Rich/Richest	0.55***	0.21**	0.13
	(0.073)	(0.077)	(0.086)
**Wealth & Education interaction**			
Poorer/Middle*yrs.sch.c^a^			-0.031*
			(0.015)
Rich/Richest* yrs.sch.c			-0.027
			(0.015)
**Four or more ANC visits**	0.78***	0.60***	0.60***
	(0.051)	(0.055)	(0.055)
**First ANC** (ref = first trimester)			
Second trimester	-0.24***	-0.086*	-0.088*
	(0.041)	(0.042)	(0.042)
Third trimester	-1.01***	-0.50***	-0.50***
	(0.11)	(0.11)	(0.11)
DK trimester	-2.26***	-1.76***	-1.76***
	(0.52)	(0.50)	(0.50)
**ANC provider** (ref = Nurse			
Doctor	0.17**	0.049	0.051
	(0.054)	(0.053)	(0.053)
All others	-0.58***	-0.37**	-0.36**
	(0.14)	(0.13)	(0.13)
**ANC facility** (ref = Gov't hosp/polyclinic)			
Other Gov't facility	-0.34***	-0.23***	-0.22***
	(0.051)	(0.051)	(0.051)
Only Private facility/maternity home	-0.29***	-0.31***	-0.31***
	(0.065)	(0.061)	(0.061)
Home/other/DK	-2.20***	-1.84***	-1.85***
	(0.21)	(0.21)	(0.21)
**Serious complication**	0.14**	0.21	0.22
	(0.053)	(0.11)	(0.11)
**Marital Status** (ref = Currently married)			
Cohabiting	-0.25***	-0.16*	-0.16**
	(0.063)	(0.062)	(0.062)
Previously married	-0.048	-0.023	-0.029
	(0.079)	(0.081)	(0.081)
Never married	-0.033	-0.014	-0.011
	(0.080)	(0.087)	(0.087)
**Ever contraception**	0.33***	0.21***	0.21***
	(0.046)	(0.046)	(0.046)
**Know family planning source**	0.14***	0.14***	0.14***
	(0.041)	(0.039)	(0.039)
**Religion** (ref = Orthodox Christian) ^b^			
Other Christian.	-0.033	-0.026	-0.026
	(0.051)	(0.049)	(0.049)
Moslem	0.026	0.20*	0.19*
	(0.077)	(0.079)	(0.080)
Traditionalist /other	-0.28***	-0.022	-0.013
	(0.083)	(0.080)	(0.080)
**Ethnicity** (ref = Akan)			
Ga/Dangme/Guan	-0.21*	-0.040	-0.045
	(0.085)	(0.081)	(0.081)
Ewe	-0.29***	-0.096	-0.099
	(0.085)	(0.082)	(0.082)
Mole-Dagbani/Hausa	-0.32***	-0.19	-0.19
	(0.096)	(0.10)	(0.10)
Grussi/Gruma	-0.31**	-0.14	-0.14
	(0.098)	(0.097)	(0.097)
Other/4missing	-0.33***	-0.21*	-0.21*
	(0.093)	(0.099)	(0.099)
**Region** (ref = Greater Accra)			
Central	0.60*	0.58**	0.57**
	(0.26)	(0.19)	(0.19)
Western	1.32***	1.38***	1.38***
	(0.27)	(0.20)	(0.20)
Volta	-0.24	0.050	0.050
	(0.27)	(0.20)	(0.20)
Eastern	0.32	0.42*	0.41*
	(0.25)	(0.18)	(0.18)
Ashanti	0.69**	0.65***	0.65***
	(0.25)	(0.18)	(0.18)
Brong Ahafo	0.70**	0.79***	0.78***
	(0.26)	(0.19)	(0.19)
Northern	-0.44	0.010	0.0099
	(0.27)	(0.20)	(0.20)
Upper East	0.59	0.93***	0.95***
	(0.30)	(0.23)	(0.23)
Upper West	0.39	0.81***	0.83***
	(0.32)	(0.24)	(0.24)
Constant		6.37***	6.45***
		(0.22)	(0.22)
N	4868	4868	4868

Notes: *p<0.05, ** p<0.01, *** p<0.001. Standard errors in parentheses.

^a^ yrs.sch.c. = Years of school centered.

^b^ Orthodox refers to Catholic/Methodist/Presbyterian. Other Christian refers to Pentecostals, charismatics, protestants, and other Christians.

All Models also include age, parity, miscarried/stillbirth, any pregnancy complication, female headed household, age at marriage, and reason for ANC, but these are not significant even in the bivariate models. There were however left were left in the model because their exclusion increased the size of the coefficient for the other variables suggesting they may be playing a role and their exclusion will increase omitted variable bias.

The multivariate analyses with all the relevant independent variables in the model are shown in the last two columns of Table 4. The full unconditional model shows that when other factors are accounted for there is no significant rural/urban difference in ANC quality, but both education and wealth are still significantly associated with ANC quality, although the effects are small. Each additional year of education increases the ANC quality score by about 0.02 points. On average, women with some education (any level) receive higher quality ANC than those with no education (b = 0.12, p = 0.04 for primary education, b = 0.21, p<0.001 for middle/JSS and b = 0.17, p = 0.053 for secondary education or higher; the differences beyond primary education are not significant). Also, women in the higher wealth quintiles (poorer/middle and richer/richest) receive significantly higher quality ANC than those in the lowest wealth quintile (poorest), but the difference between women in the higher wealth quintiles is not significant (b = 0.043, p = 0.469).

The multivariate models also show that after accounting for other factors, number of ANCVs, trimester of the first ANCV, type of ANC facility, and type of ANC provider are still significantly associated with ANC quality. On average, women who had four or more ANCVs score 0.60 more points on ANC quality than those who had less than four visits. Women who went for the first ANCV in the first trimester also received significantly higher quality ANC than those who went later. In the adjusted model, there is no difference in the quality of ANC received from doctors and that from nurses/midwifes, but those who received ANC from an unskilled provider received significantly lower quality ANC. Net of other factors, women who received ANC in a government hospital or polyclinic received significantly higher quality ANC than those who received ANC from any other type of facility. There is no significant difference in the quality of ANC received in private facilities and that received in lower tiered government facilities. In general, ANC quality received in any health facility is higher than that received outside a health facility.

On average, women who are cohabiting are less likely to receive high quality ANC than those married. Women who have ever used contraception and women who know where to get contraception are also more likely to receive higher quality ANC than those who have never used contraception and those who do not know where to get contraception respectively. Net of other factors, Moslems received higher quality ANC than all the other religious groups. Also, women in most regions (except Volta and Northern region) received higher quality ANC than those in the Greater Accra region. Women in the Western region received the highest quality ANC (b = 0.43, p = 0.037 for Western region compared to the Upper East region, which has the next highest coefficient), but there are no differences between the other regions with better ANC quality than the Greater Accra region.

None of the pregnancy risk factors are significantly associated with quality of ANC in the adjusted models. Having a serious pregnancy complication is associated with higher quality of ANC, but this is only marginally significant (b = 0.22, p = 0.05). Age and parity are also not significant determinants of ANC quality. Gravidity is not used in the final multivariate results because of collinearity with parity; it is however not significant when used instead of parity. Collinearity diagnostics show no evidence of multicollinearity of the remaining variables in the models. The mean VIF is 1.48, with the highest VIF for any variable being 3.10 for parity. Excluding parity from the models does not change the results.

Moderation results

**Fig 1:**
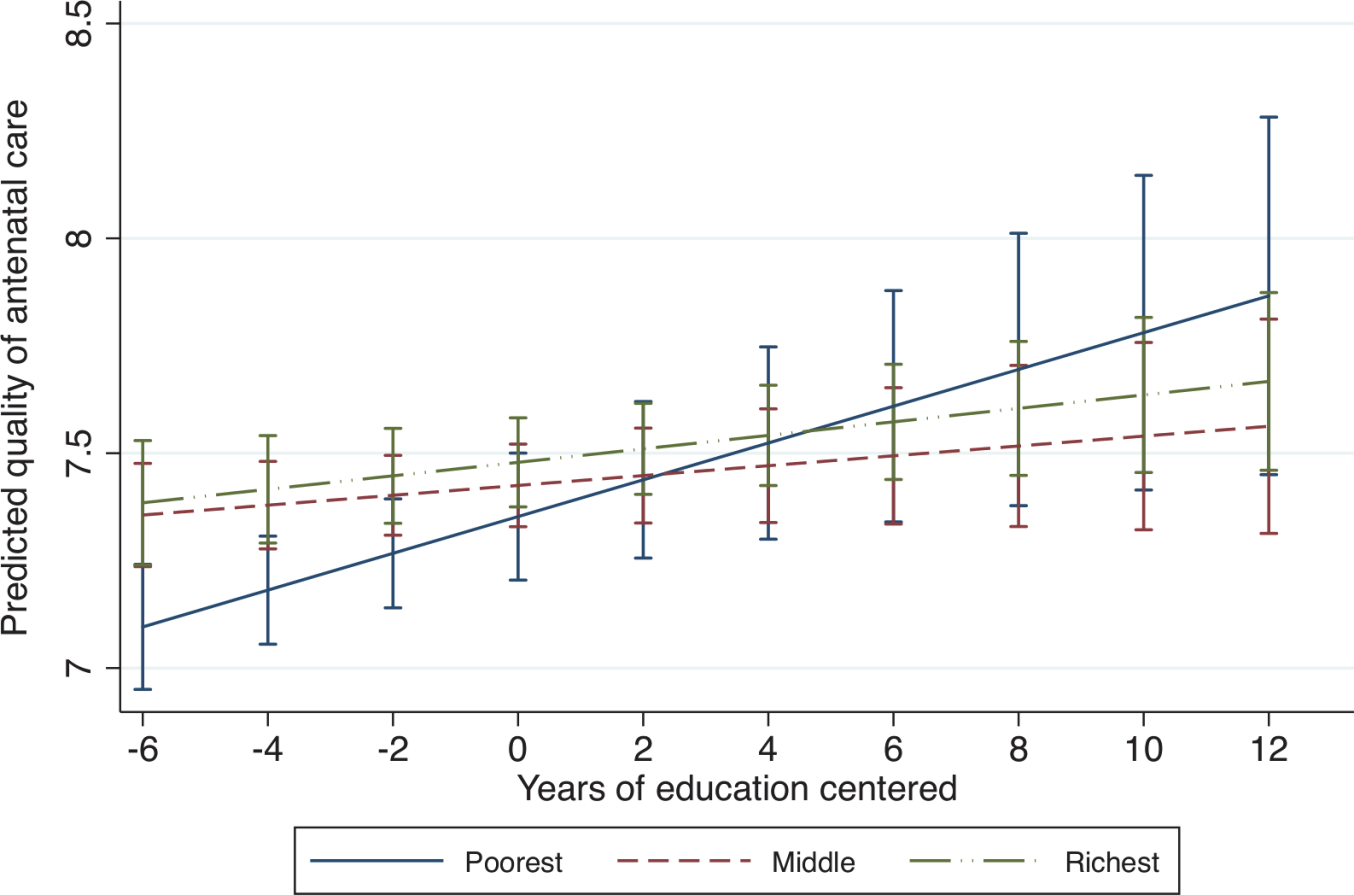
Quality of antenatal care by education and wealth. This is a graph from the interaction of education and wealth on quality of antenatal care. It shows that quality of antenatal care increases with education, but the magnitude of this change is greatest among the poorest women. The graph also shows that effect of wealth is only significant at low levels of education.

The full conditional model in Table 4 shows the interaction between education and wealth. The coefficient for education in the conditional model is the effect of education among the poorest women (the reference group for wealth). This shows that among the poorest women, each additional year of education increases the ANC quality score by about 0.04 points. The coefficients for wealth in the conditional model are the effects of wealth at the average level of education (since education is centered at the grand mean). The statistically insignificant coefficients suggest that at the average level of education there is no difference in ANC quality by wealth. The coefficients for the interaction terms are the differences in the slopes for education between women in the middle and poorest wealth quintiles and that between women in the richest and poorest wealth quintiles. They show that the magnitude of the effect of education on ANC quality among the poorest women differs from that of the middle wealth group, but not from the richest group of women. The plot of the interaction in Fig. 1 illustrates these results clearly. Fig. 1 shows the significant increase in ANC quality with education among the poorest women, and the non-significant and marginally significant increase for women in the middle and richest wealth groups respectively. We also see the difference in ANC quality by wealth at lower levels of education, but no difference by wealth at higher levels of education, including at the average level (0 years of education for the centered variable); the difference between the middle and richest wealth groups are not significant even at lower levels of education. In summary, the results from the conditional model show that poor women with no education receive the lowest quality ANC, but education is most beneficial for the poorest with regards to quality of ANC.

Mediation results

The results of the mediation analysis are shown in Table 5 (approach to mediation analysis is illustrated in S1 Fig.). The first sets of coefficients are from the unadjusted models. The partial unconditional models (PUMs) are the models with all the independent variables except one or two independent variables of interest—the potential mediating variables. PUM1 includes all independent variables except education and wealth, and the coefficient for place of residence is its total effect on ANC quality. PUM1 shows that, when SES is not accounted for, women who live in urban areas receive significantly higher quality ANC than those who live in rural areas. The coefficient for urban, however, decreases by about 58% from the bivariate model ((0.36–0.0.15)/0.36 = 0.583), suggesting more than half of the unadjusted urban effect is explained by the other independent variables in the model. When education and wealth are added to the model (full unconditional model), the coefficient for urban decreases by almost half (0.15–0.08 = 0.07) and is no longer significant. This difference is the rural/urban effect mediated by SES, and it is significant at p<0.001. The proportion of the total effect that is mediated is 0.47 (0.07/0.15 = 0.467). These results imply that close to half of the total effect of rural/urban residence on ANC quality is through SES; and rural/urban residence has no significant direct effect on ANC quality when other factors including SES are accounted for.

**Table 5:** Mediation Models-Multilevel linear regression of ANC quality on place of residence, socioeconomic status, mediating, & other variables.

		*Quality of ANC*: *b(se)*						
	Bivariate	Partial unconditional models (PUMs)						Full unconditional model
*Independent variables*		PUM1	PUM2	PUM3	PUM4	PUM5	PUM6	
**Urban residence**	0.36***	0.15*				0.11	0.13	0.084
	(0.068)	(0.061)				(0.069)	(0.068)	(0.066)
**Years of sch. centered**	0.040***		0.020***		0.018**	0.022***	0.019**	0.018**
	(0.0054)		(0.0058)		(0.0059)	(0.0060)	(0.0060)	(0.0059)
**Wealth** (ref = poorest)								
Poorer/Middle	0.26***			0.18**	0.17**	0.19**	0.17**	0.17**
	(0.062)			(0.059)	(0.059)	(0.061)	(0.061)	(0.060)
Rich/Richest	0.55***			0.27***	0.24***	0.30***	0.24**	0.21**
	(0.073)			(0.072)	(0.072)	(0.079)	(0.079)	(0.077)
**Four or more ANC visits**	0.78***	0.62***	0.62***	0.61***	0.60***		0.59***	0.60***
	(0.051)	(0.055)	(0.055)	(0.055)	(0.055)		(0.056)	(0.055)
**First ANC** (ref = first trimester)								
Second trimester	-0.24***	-0.087*	-0.086*	-0.084*	-0.085*		-0.097*	-0.086*
	(0.041)	(0.042)	(0.042)	(0.042)	(0.042)		(0.042)	(0.042)
Third trimester	-1.01***	-0.50***	-0.49***	-0.50***	-0.50***		-0.53***	-0.50***
	(0.11)	(0.11)	(0.11)	(0.11)	(0.11)		(0.11)	(0.11)
DK trimester	-2.26***	-1.77***	-1.76***	-1.77***	-1.76***		-1.95***	-1.76***
	(0.52)	(0.50)	(0.50)	(0.50)	(0.50)		(0.51)	(0.50)
**ANC provider** (ref = Nurse)								
Doctor	0.17**	0.056	0.051	0.057	0.050	0.074		0.049
	(0.054)	(0.053)	(0.053)	(0.053)	(0.053)	(0.054)		(0.053)
All others	-0.58***	-0.37**	-0.38**	-0.38**	-0.37**	-0.29*		-0.37**
	(0.14)	(0.13)	(0.13)	(0.13)	(0.13)	(0.14)		(0.13)
**ANC facility** (ref = Gov't hosp/p.clinic)								
Other Gov't facility	-0.34***	-0.25***	-0.26***	-0.24***	-0.24***	-0.23***		-0.23***
	(0.051)	(0.051)	(0.050)	(0.050)	(0.050)	(0.052)		(0.051)
Only Private/maternity home	-0.29***	-0.30***	-0.31***	-0.31***	-0.31***	-0.30***		-0.31***
	(0.065)	(0.061)	(0.061)	(0.061)	(0.061)	(0.063)		(0.061)
Home/other/DK	-2.20***	-1.86***	-1.86***	-1.86***	-1.85***	-1.93***		-1.84***
	(0.21)	(0.21)	(0.21)	(0.21)	(0.21)	(0.21)		(0.21)
N	4868	4868	4868	4868	4868	4868	4868	4868

Notes: *p<0.05, ** p<0.01, *** p<0.001. Standard errors in parentheses; All the multivariate models include the set of independent variables shown in Table 4 except

PUM 1 also excludes education and wealth; PUM 2 place of residence and wealth; PUM 3 excludes place of residence and education; PUM 4 excludes place of residence; PUM 5 excludes number of ANC visits and trimester of first visit; PUM 6 excludes ANC facility and provider.

PUM 2 includes all the independent variables except place of residence and wealth, and PUM3 includes all the independent variables except place of residence and education. These models show significant effects of both education and wealth net of other variables. PUM 4 includes all independent variables except place of residence. The change in the coefficients for both education and wealth from PUM2 and PUM3 to PUM4 (their indirect effects) are very small, but significant (p = 0.002). These results imply, a small amount of the effect of education is through wealth, and the reverse, but a larger amount of their effects are independent of each other. The full unconditional model also shows a significant direct effect of education and wealth when place of residence is added to the model. The coefficients for education and the poorer/middle wealth quintile do not change from PUM4; and the coefficient for the richer/richest wealth quintile decreases by 13% (0.24–0.21/0.24 = 0.125), but this change is not significant (p = 0.206). These results imply that the SES difference in ANC quality is not mediated by place of residence. The mediation analysis therefore supports the hypotheses that SES partially explains the rural/urban differences in ANC quality, but the reverse is not true—place of residence does not explain the SES difference in ANC quality.

In PUM5 and PUM6, I examine how much of the rural/urban and SES effects are due to the frequency and timing of ANCVs, and to the type of ANC facility and provider, respectively. PUM 5 includes all the independent variables except number of ANCVs and trimester of the first ANCV; and PUM 6 includes all the independent variables except the type of ANC facility and provider. The coefficient for urban residence is not significant in both PUM 5&6. This implies that even when frequency and timing of ANCVs and the type of ANC facility and provider are not accounted for, urban residence has no significant direct effect on ANC quality net of demographic and socioeconomic factors. (The coefficient for urban is still not significant when the ANC frequency, timing, facility and provider are all excluded from the model, but it is significant in all the models when wealth and education are not in the model.) There is a decrease in the coefficient for urban from PUM5&6 to the full unconditional model from 0.11 and 0.13 to 0.084; implying 23.6% and 35.6% of the urban effect may be mediated by the timing and frequency of ANCVs and the ANC facility and provider respectively. But because the coefficients for urban in these models are all not significant, we cannot say (with at least 95% confidence) that the mediated effects are not due to chance. (The effect of rural/urban residence that is mediated by ANC timing and frequency, and ANC facility and provider are significant when wealth and education are not in the model.)

The differences in the coefficients for education and wealth from PUM5 to the final unconditional model, are their effects mediated by the frequency and timing of ANCVs. Frequency and timing of ANCVs account for about 18% of the effect of each additional year of education (0.022–0.018/ 0.022 = 0.182) on ANC quality; 11% of the quality difference between women in the poorest and middle wealth quintiles (0.19–0.17/0.19 = 0.105); and 30% of the quality difference between women in the poorest and richest wealth quintiles (0.30–0.21/0.30 = 0.30). These mediated effects are all significant (p<0.001). The differences in the coefficients for education and wealth from PUM6 to the final unconditional model are their effects mediated by the type of ANC facility and provider. The type of ANC facility and provider account for about 5% of the effect of each additional year of education (0.019-.018/0.019 = 0.053) on ANC quality and 13% of the quality difference between women in the poorest and richest wealth quintiles (0.24–0.21/0.24 = 0.125). These mediated effects are significant (p<0.01). In summary, these results suggest some of the SES differentials in ANC quality are because higher SES women start ANCVs early and have more frequent ANCVs and receive ANC in higher level facilities (government hospitals and polyclinics) and from skilled providers. But there is a significant direct effect of SES on ANC quality that is not through these factors.

### Sensitivity analysis

Prior studies based on similar ANC quality measures dichotomized the ANC quality score to create a binary outcome, and then used logistic regression analysis [20,23]. Mediation analysis is however more complicated for binary outcomes, because the addition of variables to a logistic model changes it’s scale, which makes it inaccurate to use the change in the magnitude of the coefficients as the mediated effects [38,52]. Therefore, since the distribution of the ANC quality variable in this analysis permitted its use as continuous variable, it was used as such in linear regression. Also, multilevel mediation is much more developed for continuous outcomes than categorical outcomes. Simulation studies show that though the standard errors from multilevel linear regression tend to be larger than those from single level regression; hence, a higher likelihood of finding non-significant mediated effects (type II errors); the differences are minimal [39]. But, there is less on how multilevel mediation with binary outcomes compares to that from single level logistic regression. Nonetheless, to check if the findings are consistent for different specifications of the ANC quality variable, a binary variable was created from the ANC quality score (coded 0—received zero to seven services–lower quality; and 1– received eight or nine services– higher quality), and the full models examined with multilevel logistic regressions. The findings from this analysis are consistent with that from the multilevel linear regression in terms of statistical significance and direction of the associations. Of note, however, is that the interaction between education and wealth is not significant in the logistic model.

In addition, because the weights provided with the datasets cannot be applied for multilevel analysis, the models were run using weighted single level regressions as a sensitivity check. The results from this are also similar to the unweighted multilevel analysis in the direction and significance of the associations for most variables. The effect sizes for some of the independent variables in the weighted linear regression are, however, slightly larger than that from the multilevel analysis. In addition, a few variables such as having a pregnancy complication and a serious pregnancy complication, which are not significant in the multilevel analysis, are significant in the single level analysis. This is expected, since single level analysis may underestimate standard errors when there is clustering [39,47]. The results from the single level linear regression model also shows that the independent variables in the model explain about 23% of the variation in quality of ANC (R-squared = 0.23, p<0.001). The sensitivity analyses therefore show that the findings presented are robust, and at worst underestimate the effect of some of the factors.

## Discussion

Most women in Ghana go for ANC at least once during pregnancy, but many are not (or at least do not remember) receiving the full components of ANC. The factors significantly associated with higher quality ANC (controlling for other factors and clustering) include: higher SES (education and wealth); starting ANCVs in the first trimester; attending four or more ANCVs; receiving ANC from a government hospital or polyclinic; receiving ANC from a doctor, nurse, or midwife; living in regions other than Greater Accra, Northern, and Volta regions; ever used contraception; knowledge of where to get contraception; and being Moslem. Women who are cohabiting receive lower quality ANC than those currently married. Urban residence is associated with higher quality ANC in the bivariate and partial models, but this effect is fully explained by the variables in the model. SES accounts for a significant proportion the rural/urban effect. The SES effect is partly due to early initiation of ANCVs, more frequent ANCVs, use of higher-level health facilities, and use of skilled providers. But there is a significant direct effect of SES net of these factors. Wealth and education have independent effects on ANC quality, but there is a moderation effect, with education most beneficial for the poorest.

### Factors underlying rural/urban and SES disparities in ANC quality

I expected SES and the other factors to explain some, but not all, of the rural/urban difference in quality of ANC. The quality of ANC is expected to be lower in rural areas because these areas have less developed health infrastructure and fewer well trained health workers. Qualitative studies in Ghana also suggest rural women are more likely to be treated poorly—even when they seek care in urban facilities [53,54]. Studies in Nepal and Zambia using ANC quality measures similar to those used in this study also found higher quality ANC in urban areas [20,23]. A potential reason for the disparity from prior studies is that some of these studies controlled for fewer variables or did not control for important mediating variables like frequency and timing of ANCVs.

The positive association between SES and quality of ANC are consistent with findings from other studies on quality of primary health care services and family planning [41,42,53] as well as those from qualitative studies on quality of delivery services [54–56]. The few other quantitative studies on quality of ANC also found significant positive associations between SES and ANC quality—despite some methodological differences [19,22,23]. Possible reasons for quality of care differentials by SES include hypotheses that women with higher SES live in areas where quality of care is generally higher; use health facilities that offer higher quality of care; can physically access and afford high quality care; know what type of care to seek and are able to advocate for it; have higher expectations of care and insist on it; and are more likely have a relationship with health personnel, which helps them acquire high quality services. The narrower social power gap between high SES women and health personnel may also allow higher status women to assert their preferences to obtain high quality care [12,13,41,42,53,57]. For quality of ANC based on services received, the differential in quality of care by SES may also be due to differential use of ANC services. These hypotheses have generally not been empirically examined, or even critically evaluated, because data on most of the mediating factors are usually not available. Nevertheless, by carefully examining the associations, and how they vary in the presence of other factors, we may be able to tell which factors are predominant.

First, that SES explains the rural/urban difference in ANC quality, and not the reverse, reduces support for the hypotheses that high SES women receive better quality of care because of where they live. This does not mean the quality of care in one’s place of residence is unimportant; it means a woman’s SES, if high, may enable her to obtain better quality of care above what is available in her immediate community. Women of higher SES are not limited to seeking care where they live: they can go outside of their communities to access higher quality care. Women are willing to travel long distances to seek better health care if it is within their means [12,56]. The finding may also mean that the quality of care received by women in the same communities, and potentially within the same health facilities, is not uniform; such that women of higher SES are able to obtain higher quality care than lower SES women in the same communities and facilities. This can be because higher SES women know what services are needed, and ask for these services. Higher quality care may also be more financially accessible to higher SES women. For example, women may have to pay for lab tests during ANCVs, and poorer women in the same facility may not get this service because of costs. This was the case in Ghana until 2007 when a free maternal health policy was introduced. Since the implementation of the policy was very slow, most women who participated in the GMHS were subject to some financial cost for antenatal services [58,59].

Second, if we assume that most of the effect of education is through knowledge of what to receive and being able to ask for it, and that of wealth mostly through financial access. Then the independent effects of education and wealth on ANC quality suggest independent roles of knowledge, assertiveness, and financial access. But the steep increase in quality of ANC with education among the poorest women, with little effect of education among those in the higher wealth groups, also suggest knowledge and assertiveness are more important for the poorest women. A reason for this may be that, in some facilities and among some providers, certain services may be offered to all wealthier women, but not to poorer women, assuming that they cannot pay. Education becomes important in this instance because, poor educated women may be more likely to know what to expect, and so can actively seek it. Poor women with no education are therefore most vulnerable: they may not be offered certain services because it is assumed they cannot pay for it, and they do not ask for it because they do not know they are required to receive it. Also, because of the wide power gap between health providers and poor women with no education, they may be unable to assert their preferences even if they know what to ask for [53]. On the other hand, the higher effect of wealth at very low levels of education suggests wealthier women use facilities and providers that provide higher quality care, and so it does not require any effort on their part to receive higher quality care. In this case, educated but poor women may know where to seek high quality, but affordable care. The non-significant interaction of education and wealth in the binary logistic regression (in sensitivity analysis) is potentially because the buffering by education is important for small changes in quality, but unimportant for bigger changes—whether one received the highest quality of care or not—as cost may be a bigger barrier to receiving the best care.

Third, the findings support the hypotheses that women of higher SES receive higher quality of care partly because they use facilities and providers that provide higher quality care.

Higher SES women are more likely to receive ANC from government hospitals and polyclinics, where ANC quality is higher. They are also more likely to receive ANC from doctors, who may provide higher quality ANC. Higher SES women are also more likely to receive ANC from private health facilities. In this study, ANC quality in private facilities was the same as that in health centers and other lower level government facilities, but lower than that in government hospitals and polyclinics. But other studies suggest quality of care is higher in private facilities, though this is more for interpersonal than technical quality [42,57,60,61]. The findings therefore suggest some selection by education and wealth into facilities that provide, or are thought to provide, higher quality care. The significant mediated effect of education and wealth on ANC quality through the type of ANC facility and provider provides further support for this hypothesis. The small size of the mediated effect may be due to the opposite effects of the facilities (government hospitals and private facilities) that more educated and wealthier women use. (To explore these effects further, I examined wealth and type of ANC facility and provider interactions to see if the effects of ANC facility and provider were conditional on wealth, but these were not significant.)

Fourth, the mediation analysis with frequency and timing of ANC shows that some, but not all, of the SES effect is due to differential use of ANC services. Women who go for the first ANCV in the first trimester and attend the recommended number of ANCVs receive higher quality ANC. This may be because, even though the ANC quality measure only captures basic services that can be provided during the first ANCV, the services may not be available at all times, such that, those who start early and go more frequently have a higher chance of receiving them. Higher SES women are also more likely to start ANCVs early and attend more frequently, partly because the services are more accessible to them [12,13,20,23]. Thus, if the entire SES difference in ANC quality was explained by differential utilization, then the SES difference could be attributed mostly to differential access. The results however show that though frequency and timing of ANCVs account for some of the SES difference in ANC quality, there is a direct effect of SES net of these factors. This implies factors other than those related to reaching ANC sites (as discussed above) are also contributing to the SES difference in ANC quality. This finding is important because, it suggests factors operating within the health system may be causing the disparities in quality of ANC. The few prior studies on quality of ANC have, however, not made this distinction.

For example, in Nepal, Joshi et al found higher education, higher wealth, and urban residence to be associated with higher quality ANC (based on a similar measure of ANC quality) and higher frequency of ANCVs [23]. But they did not include frequency and timing of ANCVs as predictors of ANC quality. Tran et al in Vietnam also found a positive association between education and wealth and ANC adequacy (created from a combination of frequency, timing, and content of ANCVs); thus, did not account for the effect of the timing and frequency on the content of ANC [22]. This analysis was stratified by rural and urban, and so did not assess rural/urban differences, and wealth was only significant for rural areas. Because frequency and timing of ANC were not controlled for in these studies, it is unclear how much of the SES and rural/urban differences in quality of ANC were due to differential utilization of ANC. Also, combining frequency, timing, and content of ANCVs into a composite measure does not enable identification of the potentially different underlying reasons for differences in each of those factors.

### Other determinants of ANC quality

One other notable finding in this analysis is that, on average, women in all regions of Ghana (except Volta and Northern regions) received higher quality ANC than those in the Greater Accra region; and women in the Western region received the highest quality ANC. This is unexpected because, the Greater Accra region, which houses Accra—the national capital of Ghana—has one of the two largest teaching hospitals in the country, and has more health facilities and health personnel than any other region in the country [62,63]. Utilization of maternal health services including use of skilled birth attendants is also higher than that in the other regions [28]. Furthermore, it is more urban, hence has greater ease of reaching health facilities, and has a larger proportion of high SES women [28]. The lowest quality of ANC in Northern region can be easily explained: it has the lowest density of health workers and health facilities in Ghana, and tend to have the poorest maternal health indicators in the country [28,62,63]. On the other hand, that Western region has the highest quality ANC is more difficult to explain, considering that it has the second lowest density of health workers in the country [63]. A potential reason for the higher quality ANC in the other regions than Greater Accra region is that: because access to health services are worse in the other regions, those who go for ANCVs in these regions are a select group who are able to overcome the barriers to receiving better quality care. This is however not very likely since over 95% of pregnant women in all regions go for ANC at least once. Another potential reason is the large number of private health facilities in the Greater Accra region, which may be providing less than optimal ANC. This is plausible considering that Western, Upper East, and Upper West regions, which have fewer private facilities, appear to be doing better. The lower quality of ANC in Accra may also point to poor quality ANC in the peripheral health facilities, which are overshadowed by the presence of the teaching hospital in the region. This is plausible considering the number of mismanaged cases that are referred to the teaching hospital. To my knowledge no study has examined regional variations on quality of ANC in multivariate analysis for Ghana, thus there are no studies to compare. This finding presents an area for further studies to identify the factors that account for the regional variations in ANC quality in Ghana.

The positive effects of use of contraception and knowledge of where to get contraception on ANC quality are likely due to familiarity with the health system. But it is unclear why Moslem women receive higher quality ANC than Christians, when other factors are controlled for, and why women who are cohabiting receive lower quality ANC than those currently married. A possible reason is that Moslem women are more able to advocate for themselves for better quality care, but this effect is suppressed because of their lower SES, and only emerges when we account for SES. For the effect of cohabitating, a potential reason is stigmatization in a country where unmarried women who are pregnant are often frowned upon. This explanation is however more plausible for interpersonal quality of care, which is not adequately captured by the measure of ANC quality used in this analysis. Prior studies on quality of ANC have not adequately examined marital status and religion; those that did, found no significant effects of marital status and religion on quality of ANC [19,64]. Further studies are needed to understand the findings from this analysis.

### Limitations and strengths

There are a number of limitations to this study. First, the measure of quality of ANC only captures service provision. The questions in the GMHS are useful for evaluating whether or not women are receiving the essential ANC services, but they do not capture the experience of women with the health system—how they are treated and the nature of the interactions with health providers. Until recently, quality of maternal health care, and particularly patient experience of care, has received relatively little attention in most of SSA [5]. Data on patient experience are not collected in the surveys that serve as the major sources of maternal health data in SSA. This is potentially because there are no well validated instruments that can be easily incorporated into these surveys. Quantitative data on patient experience are however important, because qualitative studies suggest that poor attitudes of health workers are a major barrier to use of maternal health services. These studies have also suggested differential patient experience of care by SES and place of residence [53–56], which may even be bigger than those related to services received.

The measure of quality of ANC also has some limitations even as a measure service provision. For instance, we expect it to capture some dimensions of structure (human and physical resources) and process (mostly the technical aspects), as a minimum of these is required to provide services. But the questions asked are limited in discriminating between basic and more advanced infrastructure and technical expertise. They are also insufficient to determine if women who had various screening tests were adequately followed up and appropriately managed. For example, there are instances where women present in labor with the following: severe anemia because a diagnosis of mild anemia early in the pregnancy was never reassessed and managed; sickling crises because a positive sickling test during ANC was never followed up with a hemoglobin electrophoresis (to confirm sickle cell disease or sickling trait); or eclampsia because a moderately high blood pressure was not reassessed and managed. A blood sample may also be used for one test without running other essential tests (e.g., an HIV test, but not a hemoglobin test). The current measure of quality of ANC will however group all these women as having received high quality ANC. Thus, asking if a woman had the services listed at least once during pregnancy is limited in discriminating between different levels of quality. These limitations are however difficult to address in the absence of clinical assessments and documentation, paired with maternal recall. Thus, the limitations discussed should not undermine the findings presented here, but they suggest that the high score on the quality index should not be taken as an indication of high quality of ANC in Ghana; and the magnitude of the disparities in the quality of ANC are potentially larger than shown in this analysis. The limitations also call for greater efforts to develop, and validate better measures of quality of antenatal (and also delivery) care—which capture both service provision and patient experience—and incorporate them into the major health surveys in developing countries.

Another limitation of the study is that it is based on cross-sectional data, which limits causal inference. Recall and social desirability bias are also potential limitations, since the data are based on self-report. The period of recall, which may be up to five years for some, could affect the precision of reporting the services received. Other studies have however suggested women have relatively good recall of maternal events in this time window [19,65]. On the problem of social desirability, women may report they received the services because they know they are expected to have received them, which may lead to overestimation of the quality of care. This may also be more likely for some groups of women than others. The omission of variables that are related to the dependent and key independent variables from the analysis may lead to omitted variable bias, hence problems of endogeneity and unobserved heterogeneity [38,45]. Even though several factors were controlled for, this cannot be completely ruled out. The other source of endogeneity—simultaneity or reverse causation—is less of a problem for the focal relationships, as it is highly unlikely that ANC quality will lead to one’s education, wealth, or place of residence. The reverse is however more plausible, which increases confidence in causal inference based on the temporal ordering of the events. Problems of simultaneity may be bigger if one is estimating the effect of variables such as frequency of ANCVs, as the quality of care one receives during an initial visit may influence the decision to go for subsequent visits [19]. Further analysis on a restricted sample—women who attended at least four ANCVs—however showed that the potential endogeneity of ANCVs has little effect on the key relationships.

This study has several strengths. First, it addresses a gap in the maternal health literature, which is the dearth of quantitative studies on the determinants of quality of maternal health services in SSA. Second, it uses a nationally representative sample of women who had a birth (live or stillbirth) in the five years preceding the survey, hence has high generalizability. Unlike analysis based on the usual DHS, which will include only women with a live birth (the group that are asked the maternal health questions), the inclusion of all women with a birth in the preceding five years reduces the chances of excluding women who received the worst care, as these women may be more likely to have a stillbirth. The restriction of the sample to women that went for at least one ANCV, though necessary for the analysis, may reduce the generalizability of the findings. But this represents over 95% of women in Ghana. In addition, most of the factors that predict the quality of ANC also predict having at least one ANCV. Thus, the effects found in this study may be larger in a sample that also includes women who did not have any ANC. The analysis also uses rigorous methods and the sensitivity analysis shows the findings are robust.

## Conclusions

This study finds that majority of women interact with the health system at least once during pregnancy, but the quality of ANC they receive is suboptimal, especially for women of low SES. Differential utilization of ANC services accounts for some of the SES disparities in ANC quality, but there is a significant effect of SES net of ANC utilization. Health system factors partly account for the SES disparities in ANC quality, including differential quality of care in different types of health facilities and potentially within health facilities for different groups of women. These findings have a number of implications. First, efforts to encourage women to start ANCVs early and attend the recommended number of times should be continued. But there is greater need to increase the quality of the interaction that women have with the health system during pregnancy: through efforts to provide high quality ANC to all pregnant women as well as targeted efforts to ensure poor illiterate women are also receiving high quality ANC. Since low SES women are more likely to use the health centers and other lower level health facilities, improving the quality of care provided in these facilities will help reduce the SES disparities in quality of care. A first step towards this will be to provide the basic equipment needed to provide the essential ANC services in these facilities. A second step is refresher trainings for providers at these facilities: to remind them of the essential components of ANC, why they need to provide specific antenatal services, and how they can provide the services efficiently. Effective monitoring and supervision to ensure the right things are being done should follow these activities. Monitoring and supervision should also be extended to private facilities to ensure they are providing effective ANC. In addition, there should be efforts within health facilities to ensure low SES women are not receiving lower quality of care because of cost, lack of knowledge, lack of assertiveness, or other reasons. These interventions should be paired with efforts to attract, retain, and motivate qualified personnel in the lower level health facilities. This analysis also adds voice to calls to improve women’s SES, especially through education. Finally, that many women are not receiving optimal ANC suggests that this may be contributing to the low use of skilled birth attendants and poor maternal outcomes, despite the high antenatal attendance. The SES disparities in quality of ANC may also be contributing to the SES disparities in the use of skilled birth attendants. These hypotheses require further studies. Nonetheless, targeted efforts to increase ANC quality will help improve maternal health and reduce maternal health disparities in Ghana and SSA.

## Supporting Information

S1 Fig Illustrating approach to mediation analysis using the difference of coefficients method. (DOCX)

S1 Table Sample Distribution, Ghana Maternal Health Survey. (DOCX)

S2 Table Cross tabulation of all study variables by mean quality of antenatal care (DOCX)

S3 Table Cross tabulation of key study variables by place of residence, education, and wealth. (DOCX)
