# Supplementary material for: Rural/Urban and Socioeconomic Differentials in Quality of Antenatal Care in Ghana
Source: PLoS One. 2015 Feb 19;10(2):e0117996. doi: 10.1371/journal.pone.0117996 (PMC4335004; doi:10.1371/journal.pone.0117996)
Supplement: S1 Fig — (DOCX) [file pone.0117996.s001.docx]

**Figure S1:**

**Illustrating approach to mediation analysis using the difference of coefficients method**

**Key independent variable (s)**

**Dependent variable**

*c (total effect)*

Step 1

**Figure S1A: General approach**

**Other independent variables**

Step 2

*c’ (direct effect)*

**Dependent variable**

**Key independent variable (s)**

**Other independent variables**

**Mediating variable(s)**

***Mediated effect = total effect minus the direct effect (c-c’)***

Figure S1A shows that the mediation analysis using the difference of coefficients (c-c’) method involves two main steps. This first step is to estimate the coefficient for the key independent variable in the model with all relevant independent variables, except the potential mediating variable(s). The coefficient for the key independent variable in this model is its *total effect* referred to as c in Mackinnon’s notation. We then add the potential mediating variable(s) to obtain the full model. The coefficient for the key independent variable in the full model is its *direct effect*, referred to as c’ in Mackinnon’s notation. The value of c should be significant to suggest the key relationship is not spurious or redundant. However, it should reduce with the addition of a mediating variable to suggest a mediation effect. An insignificant c’ will suggest total mediation and a significant c’ will suggest partial mediation. The total effect minus the direct effect (c-c’) gives the *mediated or indirect effect*. The likelihood ratio test is used to test if the mediated effect is significant. The magnitude of the mediated effect can be assessed by calculating the proportion of the total effect that is mediated (mediated/total effect = (c-c’)/c = 1 – c’/c) [1,2].

**Figure S1B: Examining if the rural/urban effect on ANC quality is mediated by socioeconomic status**

*c’ (direct effect)*

**ANC quality**

**Place of residence**

**Other independent variables**

**Education,**

**Wealth**

**Figure S1C: Examining if the rural/urban and SES effects on ANC quality are mediated by ANC timing, frequency, facilities, and providers**

*c’ (direct effect)*

**ANC quality**

**Place of residence, education, wealth**

**Other independent variables**

**ANC frequency and timing**

**ANC facility and provider**

Figures S1B&C show the second steps for the mediation analysis done in this study. The first step is similar to that shown in the general approach. In this study, the mediation analysis is done with three key independent variables: rural/urban residence, education, and wealth; with quality of ANC the as the dependent variable. However, each of these is also examined as a mediator for the effect of the others. For example, to examine if some of the effect of place of residence on ANC quality is through SES, place of residence is the key independent variable and education and wealth are the mediating variables (Figure S1B). For this analysis, I start with rural/urban (bivariate model) and then introduce all other variables, except education and wealth. This is the first partial unconditional model (PUM1). I then add education and wealth to obtain the full unconditional model. The difference in the coefficients in the full unconditional model (c’) and the partial model (c) gives the rural urban effect mediated by SES. A similar approach is used to examine if the SES effect is through place of residence, with education and wealth as the key independent variables and place of residence as the mediating variable

Figure S1C shows the mediation to examine if the effect of place of residence and socioeconomic status are through the timing and frequency of ANCVs and the type of ANC facility and provider. Here, the partial models are the models with all the relevant independent variables except the timing and frequency of ANCVs, and the type of ANC facility and provider – PUM5 and PUM6 respectively. I then add each group of variables to obtain the full unconditional model. The difference in the coefficients for place of residence, education, and wealth in the full unconditional model (c’) and the partial models (c) gives their effects mediated by the timing and frequency of ANCVs, and the type of ANC facility and provider, respectively.

1. MacKinnon DP (2008) Introduction to statistical mediation analysis. New York: Lawrence Erlbaum Associates.

2. Aneshensel CS (2013) Theory-based data analysis for the social sciences. 2nd ed. Thousand Oaks, Calif.: SAGE Publications, Inc.
