# Supplementary material for: Rural/Urban and Socioeconomic Differentials in Quality of Antenatal Care in Ghana
Source: PLoS One. 2015 Feb 19;10(2):e0117996. doi: 10.1371/journal.pone.0117996 (PMC4335004; doi:10.1371/journal.pone.0117996)
Supplement: S1 Table — (DOCX) [file pone.0117996.s002.docx]

| **Table S1: Sample Distribution, Ghana Maternal Health Survey (GMHS), 2007** | | | | | | | | | | | | | |
| --- | --- | --- | --- | --- | --- | --- | --- | --- | --- | --- | --- | --- | --- |
|  | **Full analytic sample, N=5,042** | | | | | |  | **Women with at least one ANC, N=4,868** | | | | | |
|  | *Unweighted* | |  | *Weighted* | | |  | *Unweighted* | |  | *Weighted* | | |
| *Variables* | N | % |  | Proportion | [95% C.I] | |  | N | % |  | Proportion | [95% C.I] | |
| **Setting** |  |  |  |  |  |  |  |  |  |  |  |  |  |
| Rural | 3,115 | 61.8 |  | 0.657 | 0.627 | 0.687 |  | 2,967 | 61.0 |  | 0.648 | 0.617 | 0.679 |
| Urban | 1,927 | 38.2 |  | 0.343 | 0.313 | 0.373 |  | 1,901 | 39.1 |  | 0.352 | 0.321 | 0.383 |
| **Region** |  |  |  |  |  |  |  |  |  |  |  |  |  |
| Greater Accra | 636 | 12.6 |  | 0.095 | 0.079 | 0.111 |  | 619 | 12.7 |  | 0.095 | 0.080 | 0.111 |
| Central | 441 | 8.8 |  | 0.099 | 0.082 | 0.116 |  | 429 | 8.8 |  | 0.099 | 0.083 | 0.116 |
| Western | 382 | 7.6 |  | 0.082 | 0.063 | 0.100 |  | 371 | 7.6 |  | 0.082 | 0.064 | 0.101 |
| Volta | 407 | 8.1 |  | 0.092 | 0.068 | 0.116 |  | 389 | 8.0 |  | 0.092 | 0.067 | 0.117 |
| Eastern | 744 | 14.8 |  | 0.116 | 0.102 | 0.130 |  | 724 | 14.9 |  | 0.117 | 0.103 | 0.131 |
| Ashanti | 855 | 17.0 |  | 0.186 | 0.162 | 0.211 |  | 837 | 17.2 |  | 0.189 | 0.165 | 0.214 |
| Brong Ahafo | 496 | 9.8 |  | 0.115 | 0.097 | 0.133 |  | 486 | 10.0 |  | 0.117 | 0.099 | 0.135 |
| Northern | 541 | 10.7 |  | 0.137 | 0.104 | 0.171 |  | 491 | 10.1 |  | 0.131 | 0.097 | 0.165 |
| Upper east | 303 | 6.0 |  | 0.047 | 0.036 | 0.057 |  | 298 | 6.1 |  | 0.048 | 0.037 | 0.058 |
| Upper west | 237 | 4.7 |  | 0.030 | 0.021 | 0.039 |  | 224 | 4.6 |  | 0.030 | 0.021 | 0.038 |
| **Highest Education** |  |  |  |  |  |  |  |  |  |  |  |  |  |
| None | 1,697 | 33.7 |  | 0.341 | 0.307 | 0.375 |  | 1,588 | 32.6 |  | 0.330 | 0.296 | 0.364 |
| Primary | 1,109 | 22.0 |  | 0.220 | 0.203 | 0.238 |  | 1,072 | 22.0 |  | 0.221 | 0.202 | 0.239 |
| Middle/JSS | 1,830 | 36.3 |  | 0.366 | 0.338 | 0.395 |  | 1,804 | 37.1 |  | 0.375 | 0.345 | 0.404 |
| Secondary/SSS/ higher | 406 | 8.1 |  | 0.072 | 0.061 | 0.083 |  | 404 | 8.3 |  | 0.075 | 0.063 | 0.086 |
| Mean years education (SD) | 5,042 | 5.1 (4.39) |  | 5.021 | 4.711 | 5.330 |  | 4,868 | 5.2 (4.39) |  | 5.130 | 4.815 | 5.444 |
| **Household wealth index** |  |  |  |  |  |  |  |  |  |  |  |  |  |
| Poorest | 1,097 | 21.8 |  | 0.214 | 0.183 | 0.245 |  | 1,024 | 21.0 |  | 0.207 | 0.177 | 0.236 |
| Poorer | 994 | 19.7 |  | 0.215 | 0.190 | 0.239 |  | 943 | 19.4 |  | 0.210 | 0.186 | 0.235 |
| Middle | 951 | 18.9 |  | 0.202 | 0.180 | 0.224 |  | 930 | 19.1 |  | 0.204 | 0.182 | 0.227 |
| Richer | 995 | 19.7 |  | 0.198 | 0.177 | 0.220 |  | 976 | 20.1 |  | 0.203 | 0.181 | 0.224 |
| Richest | 1,005 | 19.9 |  | 0.171 | 0.151 | 0.191 |  | 995 | 20.4 |  | 0.176 | 0.155 | 0.197 |
| **Household head Female** |  |  |  |  |  |  |  |  |  |  |  |  |  |
| No | 3,790 | 75.2 |  | 0.751 | 0.730 | 0.772 |  | 3,650 | 75.0 |  | 0.749 | 0.727 | 0.771 |
| Yes | 1,252 | 24.8 |  | 0.249 | 0.228 | 0.270 |  | 1,218 | 25.0 |  | 0.251 | 0.229 | 0.273 |
| **Religious affiliation** |  |  |  |  |  |  |  |  |  |  |  |  |  |
| Catholic | 686 | 13.6 |  | 0.136 | 0.115 | 0.157 |  | 661 | 13.6 |  | 0.136 | 0.115 | 0.158 |
| Methodist/Presbyterian | 662 | 13.1 |  | 0.140 | 0.124 | 0.157 |  | 652 | 13.4 |  | 0.144 | 0.127 | 0.161 |
| Pentecostal/Charismatic | 1,476 | 29.3 |  | 0.280 | 0.261 | 0.299 |  | 1,444 | 29.7 |  | 0.283 | 0.264 | 0.302 |
| Other Christian | 832 | 16.5 |  | 0.167 | 0.151 | 0.184 |  | 810 | 16.6 |  | 0.168 | 0.152 | 0.185 |
| Moslem | 886 | 17.6 |  | 0.183 | 0.149 | 0.217 |  | 863 | 17.7 |  | 0.183 | 0.150 | 0.217 |
| Traditional/other | 500 | 9.9 |  | 0.093 | 0.075 | 0.112 |  | 438 | 9.0 |  | 0.085 | 0.069 | 0.101 |
| **Table S1 continued** |  |  |  |  |  |  |  |  |  |  |  |  |  |
| **Ethnicity** |  |  |  |  |  |  |  |  |  |  |  |  |  |
| Akan | 2,238 | 44.4 |  | 0.463 | 0.425 | 0.501 |  | 2,197 | 45.1 |  | 0.471 | 0.432 | 0.509 |
| Ga/Dangme/Guan | 521 | 10.3 |  | 0.091 | 0.071 | 0.112 |  | 504 | 10.4 |  | 0.092 | 0.071 | 0.112 |
| Ewe | 641 | 12.7 |  | 0.121 | 0.098 | 0.144 |  | 615 | 12.6 |  | 0.120 | 0.097 | 0.143 |
| Mole-Dagbani/Hausa | 604 | 12.0 |  | 0.130 | 0.095 | 0.165 |  | 583 | 12.0 |  | 0.131 | 0.096 | 0.166 |
| Grussi/Gruma | 580 | 11.5 |  | 0.109 | 0.076 | 0.141 |  | 534 | 11.0 |  | 0.103 | 0.072 | 0.134 |
| Other/4missing | 458 | 9.1 |  | 0.086 | 0.064 | 0.107 |  | 435 | 8.9 |  | 0.084 | 0.063 | 0.105 |
| **Age in years** |  |  |  |  |  |  |  |  |  |  |  |  |  |
| 15-19yrs | 247 | 4.9 |  | 0.049 | 0.042 | 0.056 |  | 236 | 4.9 |  | 0.049 | 0.042 | 0.056 |
| 20-24 | 915 | 18.2 |  | 0.183 | 0.169 | 0.196 |  | 891 | 18.3 |  | 0.185 | 0.171 | 0.198 |
| 25-29 | 1,176 | 23.3 |  | 0.229 | 0.216 | 0.243 |  | 1,138 | 23.4 |  | 0.230 | 0.216 | 0.244 |
| 30-34 | 1,115 | 22.1 |  | 0.225 | 0.213 | 0.237 |  | 1,082 | 22.2 |  | 0.226 | 0.213 | 0.238 |
| 35-39 | 913 | 18.1 |  | 0.182 | 0.171 | 0.194 |  | 881 | 18.1 |  | 0.182 | 0.171 | 0.194 |
| 40-49yrs | 676 | 13.4 |  | 0.132 | 0.121 | 0.142 |  | 640 | 13.2 |  | 0.128 | 0.118 | 0.139 |
| Mean (SD) | 5,042 | 30.5(7.37) |  | 30.487 | 30.242 | 30.732 |  | 4,868 | 30.5 (7.34) |  | 30.426 | 30.183 | 30.670 |
| **Marital status** |  |  |  |  |  |  |  |  |  |  |  |  |  |
| Currently married | 3,633 | 72.1 |  | 0.718 | 0.697 | 0.738 |  | 3,510 | 72.1 |  | 0.718 | 0.697 | 0.739 |
| Cohabiting | 687 | 13.6 |  | 0.141 | 0.125 | 0.156 |  | 666 | 13.7 |  | 0.141 | 0.125 | 0.157 |
| Previously married | 364 | 7.2 |  | 0.071 | 0.062 | 0.079 |  | 347 | 7.1 |  | 0.070 | 0.061 | 0.078 |
| Never married | 358 | 7.1 |  | 0.071 | 0.062 | 0.079 |  | 345 | 7.1 |  | 0.071 | 0.062 | 0.080 |
| **Age at first union ^a^** |  |  |  |  |  |  |  |  |  |  |  |  |  |
| Less than 19years | 2,445 | 48.5 |  | 0.493 | 0.473 | 0.514 |  | 2,337 | 48.0 |  | 0.488 | 0.467 | 0.509 |
| 19 or more years | 2,239 | 44.4 |  | 0.436 | 0.416 | 0.455 |  | 2,186 | 44.9 |  | 0.441 | 0.421 | 0.461 |
| Never in a union | 358 | 7.1 |  | 0.071 | 0.062 | 0.079 |  | 345 | 7.1 |  | 0.071 | 0.062 | 0.080 |
| Mean (SD) | 4,684 | 18.9(4.03) |  | 18.808 | 18.614 | 19.002 |  | 4,523 | 19.0 (4.03) |  | 18.871 | 18.674 | 19.069 |
| **No. of pregnancies (Gravidity)** | |  |  |  |  |  |  |  |  |  |  |  |  |
| 1-2 | 1,669 | 33.1 |  | 0.323 | 0.307 | 0.339 |  | 1,629 | 33.5 |  | 0.327 | 0.311 | 0.343 |
| 3-4 | 1,549 | 30.7 |  | 0.312 | 0.297 | 0.327 |  | 1,506 | 30.9 |  | 0.316 | 0.301 | 0.332 |
| 5plus | 1,824 | 36.2 |  | 0.365 | 0.348 | 0.382 |  | 1,733 | 35.6 |  | 0.357 | 0.340 | 0.374 |
| Mean (SD) | 5,042 | 4.0(2.44) |  | 3.989 | 3.903 | 4.075 |  | 4,868 | 3.9 (2.42) |  | 3.945 | 3.864 | 4.026 |
| **No. of children born (Parity)** |  |  |  |  |  |  |  |  |  |  |  |  |  |
| No children born alive | 22 | 0.4 |  | 0.004 | 0.002 | 0.006 |  | 19 | 0.4 |  | 0.004 | 0.002 | 0.005 |
| 1-2 | 2,072 | 41.1 |  | 0.405 | 0.388 | 0.421 |  | 2,028 | 41.7 |  | 0.410 | 0.393 | 0.427 |
| 3-4 | 1,513 | 30.0 |  | 0.303 | 0.289 | 0.317 |  | 1,471 | 30.2 |  | 0.306 | 0.292 | 0.320 |
| 5plus | 1,435 | 28.5 |  | 0.289 | 0.272 | 0.306 |  | 1,350 | 27.7 |  | 0.280 | 0.264 | 0.296 |
| Mean (SD) | 5,042 | 3.5 (2.26) |  | 3.509 | 3.419 | 3.599 |  | 4,868 | 3.4 (2.23) |  | 3.458 | 3.374 | 3.543 |
| **Table S1continued** | |  |  |  |  |  |  |  |  |  |  |  |  |
| **Ever used contraception** |  |  |  |  |  |  |  |  |  |  |  |  |  |
| No | 1,899 | 37.7 |  | 0.385 | 0.356 | 0.414 |  | 1,780 | 36.6 |  | 0.374 | 0.345 | 0.403 |
| Yes | 3,143 | 62.3 |  | 0.615 | 0.586 | 0.644 |  | 3,088 | 63.4 |  | 0.626 | 0.597 | 0.655 |
| **Know family planning source** |  |  |  |  |  |  |  |  |  |  |  |  |  |
| No | 2,376 | 47.1 |  | 0.470 | 0.447 | 0.494 |  | 2,270 | 46.6 |  | 0.465 | 0.441 | 0.489 |
| Yes | 2,666 | 52.9 |  | 0.530 | 0.506 | 0.553 |  | 2,598 | 53.4 |  | 0.535 | 0.511 | 0.559 |
| **Ever had stillbirth/miscarriage** |  |  |  |  |  |  |  |  |  |  |  |  |  |
| No | 4,000 | 79.3 |  | 0.800 | 0.787 | 0.814 |  | 3,853 | 79.2 |  | 0.799 | 0.785 | 0.813 |
| Yes | 1,042 | 20.7 |  | 0.200 | 0.186 | 0.213 |  | 1,015 | 20.9 |  | 0.201 | 0.187 | 0.215 |
| **Sibling had a maternal death** |  |  |  |  |  |  |  |  |  |  |  |  |  |
| No | 4,956 | 98.3 |  | 0.981 | 0.977 | 0.986 |  | 4,783 | 98.3 |  | 0.981 | 0.977 | 0.985 |
| Yes | 86 | 1.7 |  | 0.019 | 0.014 | 0.023 |  | 85 | 1.8 |  | 0.019 | 0.015 | 0.023 |
| **Pregnancy complication** |  |  |  |  |  |  |  |  |  |  |  |  |  |
| No | 3,956 | 78.5 |  | 0.797 | 0.783 | 0.810 |  | 3,818 | 78.4 |  | 0.796 | 0.782 | 0.810 |
| Yes | 1,086 | 21.5 |  | 0.203 | 0.190 | 0.217 |  | 1,050 | 21.6 |  | 0.204 | 0.190 | 0.218 |
| **Serious preg. complication** |  |  |  |  |  |  |  |  |  |  |  |  |  |
| No | 4,149 | 82.3 |  | 0.834 | 0.822 | 0.846 |  | 3,996 | 82.1 |  | 0.832 | 0.820 | 0.844 |
| Yes | 893 | 17.7 |  | 0.166 | 0.154 | 0.178 |  | 872 | 17.9 |  | 0.168 | 0.156 | 0.180 |
| *ANC variables* |  |  |  |  |  |  |  |  |  |  |  |  |  |
| **ANC attendance** |  |  |  |  |  |  |  |  |  |  |  |  |  |
| No | 174 | 3.5 |  | 0.036 | 0.026 | 0.045 |  |  |  |  |  |  |  |
| Yes | 4,868 | 96.6 |  | 0.964 | 0.955 | 0.974 |  | 4,868 | 100.0 |  |  |  |  |
| **ANC quality of care score** |  |  |  |  |  |  |  |  |  |  |  |  |  |
| 7 or less |  |  |  |  |  |  |  | 1,901 | 39.1 |  | 0.391 | 0.364 | 0.418 |
| 8 or 9 |  |  |  |  |  |  |  | 2,967 | 61.0 |  | 0.609 | 0.582 | 0.636 |
| Mean (SD) |  |  |  |  |  |  |  | 4,868 | 7.4 (1.52) |  | 7.406 | 7.322 | 7.490 |
| **No. of ANC visits** |  |  |  |  |  |  |  |  |  |  |  |  |  |
| 1-3 visits |  |  |  |  |  |  |  | 990 | 20.3 |  | 0.202 | 0.184 | 0.221 |
| Four or more |  |  |  |  |  |  |  | 3,878 | 79.7 |  | 0.798 | 0.779 | 0.816 |
| Mean(SD) |  |  |  |  |  |  |  | 4,868 | 5.8 (2.75) |  | 5.756 | 5.626 | 5.885 |
| **Trimester of first ANC visit** |  |  |  |  |  |  |  |  |  |  |  |  |  |
| First trimester |  |  |  |  |  |  |  | 2,688 | 55.2 |  | 0.549 | 0.529 | 0.568 |
| Second trimester |  |  |  |  |  |  |  | 1,992 | 40.9 |  | 0.413 | 0.396 | 0.431 |
| Third trimester |  |  |  |  |  |  |  | 181 | 3.7 |  | 0.036 | 0.030 | 0.042 |
| Don't know |  |  |  |  |  |  |  | 7 | 0.1 |  | 0.002 | 0.000 | 0.003 |
| **Table S1continued** |  |  |  |  |  |  |  |  |  |  |  |  |  |
| **Where ANC took place** |  |  |  |  |  |  |  |  |  |  |  |  |  |
| Gov't health facility ^b^ |  |  |  |  |  |  |  | 4,119 | 84.6 |  | 0.853 | 0.829 | 0.877 |
| Gov't hospital or polyclinic |  |  |  |  |  |  |  | 2,200 | 45.2 |  | 0.453 | 0.413 | 0.492 |
| Other Gov't facility |  |  |  |  |  |  |  | 1,919 | 39.4 |  | 0.400 | 0.361 | 0.439 |
| Only Private facility/maternity home | |  |  |  |  |  |  | 703 | 14.4 |  | 0.140 | 0.116 | 0.164 |
| Home/other/DK |  |  |  |  |  |  |  | 46 | 0.9 |  | 0.007 | 0.005 | 0.010 |
| **Highest trained ANC provider** |  |  |  |  |  |  |  |  |  |  |  |  |  |
| Doctor |  |  |  |  |  |  |  | 1,006 | 20.7 |  | 0.194 | 0.176 | 0.213 |
| Nurse |  |  |  |  |  |  |  | 3,743 | 76.9 |  | 0.785 | 0.766 | 0.803 |
| All others |  |  |  |  |  |  |  | 119 | 2.4 |  | 0.021 | 0.015 | 0.026 |
| **Reason for seeking ANC** |  |  |  |  |  |  |  |  |  |  |  |  |  |
| For checkup |  |  |  |  |  |  |  | 4,044 | 83.1 |  | 0.831 | 0.817 | 0.846 |
| For a problem/9missing |  |  |  |  |  |  |  | 824 | 16.9 |  | 0.169 | 0.154 | 0.183 |
| Notes: ^a^ This is for only women who have been in a union so does not add up to the full sample  ^b^  refers to people who had some ANC from a government facility but 98% were exclusively in a government facility. | | | | | | | | | | | | | |
