# Supplementary material for: Rural/Urban and Socioeconomic Differentials in Quality of Antenatal Care in Ghana
Source: PLoS One. 2015 Feb 19;10(2):e0117996. doi: 10.1371/journal.pone.0117996 (PMC4335004; doi:10.1371/journal.pone.0117996)
Supplement: S2 Table — (DOCX) [file pone.0117996.s003.docx]

| **Table S2: Cross tabulation of all study variables by mean quality of antenatal care, GMHS, N = 4,868** | | | | | | | | | | | | | | |  |  |  |
| --- | --- | --- | --- | --- | --- | --- | --- | --- | --- | --- | --- | --- | --- | --- | --- | --- | --- |
| *Variable* | | | | N | |  | | Mean | | [95% C.1] | | | | |  |  |  |
| Overall | | | | 4,868 | |  | | 7.406 | | 7.322 | | 7.490 | | |  |  |  |
| *ANC variables* | | | |  | |  | |  | |  | |  | | |  |  |  |
| **No. of ANC visits** | | | |  | |  | |  | |  | |  | | |  |  |  |
| 1-3 visits | | | | 990 | |  | | 6.60 | | 6.41 | | 6.79 | | |  |  |  |
| Four or more | | | | 3,878 | |  | | 7.61 | | 7.54 | | 7.68 | | |  |  |  |
| **Trimester of first ANC visit** | | | |  | |  | |  | |  | |  | | |  |  |  |
| First trimester | | | | 2,688 | |  | | 7.59 | | 7.51 | | 7.68 | | |  |  |  |
| Second trimester | | | | 1,992 | |  | | 7.26 | | 7.16 | | 7.36 | | |  |  |  |
| Third trimester | | | | 181 | |  | | 6.31 | | 5.97 | | 6.66 | | |  |  |  |
| Don't know | | | | 7 | |  | | 5.97 | | 4.92 | | 7.02 | | |  |  |  |
| **Where ANC took place** | | | |  | |  | |  | |  | |  | | |  |  |  |
| Gov't health facility only/combine | | | | 4,119 | |  | | 7.44 | | 7.35 | | 7.53 | | |  |  |  |
| Gov't hospital or polyclinic | | | | 2,200 | |  | | 7.73 | | 7.65 | | 7.81 | | |  |  |  |
| Other Gov't facility | | | | 1,919 | |  | | 7.12 | | 6.96 | | 7.27 | | |  |  |  |
| Only Private facility/maternity home | | | | 703 | |  | | 7.33 | | 7.16 | | 7.49 | | |  |  |  |
| Home/other/DK | | | | 46 | |  | | 4.91 | | 3.96 | | 5.85 | | |  |  |  |
| **Highest trained ANC provider** | | | |  | |  | |  | |  | |  | | |  |  |  |
| Doctor | | | | 1,006 | |  | | 7.73 | | 7.63 | | 7.83 | | |  |  |  |
| Nurse | | | | 3,743 | |  | | 7.35 | | 7.25 | | 7.44 | | |  |  |  |
| All others | | | | 119 | |  | | 6.59 | | 6.10 | | 7.08 | | |  |  |  |
| **Reason for seeking ANC** | | | |  | |  | |  | |  | |  | | |  |  |  |
| For checkup | | | | 4,044 | |  | | 7.37 | | 7.28 | | 7.46 | | |  |  |  |
| For a problem/9missing | | | | 824 | |  | | 7.57 | | 7.45 | | 7.70 | | |  |  |  |
| *Geographic location* | | | |  | |  | |  | |  | |  | | |  |  |  |
| **Setting** | | | |  | |  | |  | |  | |  | | |  |  |  |
| Rural | | | | 2,967 | |  | | 7.24 | | 7.13 | | 7.36 | | |  |  |  |
| Urban | | | | 1,901 | |  | | 7.71 | | 7.62 | | 7.79 | | |  |  |  |
| **Region** | | | |  | |  | |  | |  | |  | | |  |  |  |
| Greater Accra | | | | 619 | |  | | 7.19 | | 6.99 | | 7.39 | | |  |  |  |
| Central | | | | 429 | |  | | 7.63 | | 7.44 | | 7.82 | | |  |  |  |
| Western | | | | 371 | |  | | 8.25 | | 8.07 | | 8.44 | | |  |  |  |
| Volta | | | | 389 | |  | | 6.73 | | 6.33 | | 7.13 | | |  |  |  |
| Eastern | | | | 724 | |  | | 7.35 | | 7.15 | | 7.55 | | |  |  |  |
| Ashanti | | | | 837 | |  | | 7.72 | | 7.56 | | 7.88 | | |  |  |  |
| Brong Ahafo | | | | 486 | |  | | 7.68 | | 7.43 | | 7.93 | | |  |  |  |
| Northern | | | | 491 | |  | | 6.67 | | 6.43 | | 6.90 | | |  |  |  |
| Upper east | | | | 298 | |  | | 7.54 | | 7.29 | | 7.78 | | |  |  |  |
| Upper west | | | | 224 | |  | | 7.27 | | 7.05 | | 7.49 | | |  |  |  |
| *Socioeconomic variables* | | | |  | |  | |  | |  | |  | | |  |  |  |
| **Highest Education** | | | |  | |  | |  | |  | |  | | |  |  |  |
| None | | | | 1,588 | |  | | 7.03 | | 6.88 | | 7.17 | | |  |  |  |
| Primary | | | | 1,072 | |  | | 7.36 | | 7.24 | | 7.47 | | |  |  |  |
| Middle/JSS | | | | 1,804 | |  | | 7.71 | | 7.61 | | 7.80 | | |  |  |  |
| Secondary/SSS/higher | | | | 404 | |  | | 7.74 | | 7.58 | | 7.89 | | |  |  |  |
| **Household wealth index** | | | |  | |  | |  | |  | |  | | |  |  |  |
| Poorest | | | | 1,024 | |  | | 6.85 | | 6.65 | | 7.05 | | |  |  |  |
| Poorer | | | | 943 | |  | | 7.26 | | 7.12 | | 7.39 | | |  |  |  |
| Middle | | | | 930 | |  | | 7.53 | | 7.41 | | 7.65 | | |  |  |  |
| Richer | | | | 976 | |  | | 7.63 | | 7.50 | | 7.75 | | |  |  |  |
| Richest | | | | 995 | |  | | 7.84 | | 7.75 | | 7.93 | | |  |  |  |
| **Household head Female** | | | |  | |  | |  | |  | |  | | |  |  |  |
| No | | | | 3,650 | |  | | 7.36 | | 7.26 | | 7.45 | | |  |  |  |
| Yes | | | | 1,218 | |  | | 7.55 | | 7.44 | | 7.66 | | |  |  |  |
| **Table S2 continued** |  |  |  | |  | |  | |  | |  | |  |  |  |  |  |
| **Religious affiliation** | | | |  | |  | |  | |  | |  | | |  |  |  |
| Catholic | | | | 661 | |  | | 7.43 | | 7.22 | | 7.64 | | |  |  |  |
| Methodist/Presbyterian | | | | 652 | |  | | 7.58 | | 7.43 | | 7.72 | | |  |  |  |
| Pentecostal/charismatic | | | | 1,444 | |  | | 7.57 | | 7.47 | | 7.67 | | |  |  |  |
| Other Christian | | | | 810 | |  | | 7.38 | | 7.24 | | 7.52 | | |  |  |  |
| Moslem | | | | 863 | |  | | 7.27 | | 7.12 | | 7.42 | | |  |  |  |
| Traditional/other | | | | 438 | |  | | 6.89 | | 6.59 | | 7.18 | | |  |  |  |
| **Ethnicity** | | | |  | |  | |  | |  | |  | | |  |  |  |
| Akan | | | | 2,197 | |  | | 7.77 | | 7.68 | | 7.86 | | |  |  |  |
| Ga/Dangme/Guan | | | | 504 | |  | | 7.10 | | 6.89 | | 7.31 | | |  |  |  |
| Ewe | | | | 615 | |  | | 7.09 | | 6.89 | | 7.30 | | |  |  |  |
| Mole-Dagbani/Hausa | | | | 583 | |  | | 6.96 | | 6.77 | | 7.16 | | |  |  |  |
| Grussi/Gruma | | | | 534 | |  | | 7.06 | | 6.73 | | 7.39 | | |  |  |  |
| Other/4missing | | | | 435 | |  | | 7.26 | | 7.05 | | 7.46 | | |  |  |  |
| *Reproductive Health variables* | | | |  | |  | |  | |  | |  | | |  |  |  |
| **Age in years** | | | |  | |  | |  | |  | |  | | |  |  |  |
| 15-19yrs | | | | 236 | |  | | 7.34 | | 7.12 | | 7.56 | | |  |  |  |
| 20-24 | | | | 891 | |  | | 7.47 | | 7.33 | | 7.60 | | |  |  |  |
| 25-29 | | | | 1,138 | |  | | 7.44 | | 7.31 | | 7.57 | | |  |  |  |
| 30-34 | | | | 1,082 | |  | | 7.38 | | 7.25 | | 7.51 | | |  |  |  |
| 35-39 | | | | 881 | |  | | 7.49 | | 7.36 | | 7.62 | | |  |  |  |
| 40-49yrs | | | | 640 | |  | | 7.20 | | 7.06 | | 7.35 | | |  |  |  |
| **Marital status** | | | |  | |  | |  | |  | |  | | |  |  |  |
| Currently married | | | | 3,510 | |  | | 7.42 | | 7.34 | | 7.51 | | |  |  |  |
| Cohabiting | | | | 666 | |  | | 7.18 | | 6.95 | | 7.41 | | |  |  |  |
| Previously married | | | | 347 | |  | | 7.52 | | 7.27 | | 7.77 | | |  |  |  |
| Never married | | | | 345 | |  | | 7.58 | | 7.39 | | 7.76 | | |  |  |  |
| **Age at first union** | | | |  | |  | |  | |  | |  | | |  |  |  |
| Less than 19years | | | | 2,337 | |  | | 7.35 | | 7.24 | | 7.46 | | |  |  |  |
| 19 or more years | | | | 2,186 | |  | | 7.44 | | 7.35 | | 7.53 | | |  |  |  |
| Never in a union | | | | 345 | |  | | 7.58 | | 7.39 | | 7.76 | | |  |  |  |
| **No. of children ever born (Parity)** | | | | | |  | |  | |  | |  | | |  |  |  |
| No children born alive | | | | 19 | |  | | 7.26 | | 6.52 | | 8.01 | | |  |  |  |
| 1-2 | | | | 2,028 | |  | | 7.51 | | 7.41 | | 7.61 | | |  |  |  |
| 3-4 | | | | 1,471 | |  | | 7.39 | | 7.28 | | 7.50 | | |  |  |  |
| 5plus | | | | 1,350 | |  | | 7.28 | | 7.15 | | 7.40 | | |  |  |  |
| **Ever used contraception** | | | |  | |  | |  | |  | |  | | |  |  |  |
| No | | | | 1,780 | |  | | 7.01 | | 6.88 | | 7.15 | | |  |  |  |
| Yes | | | | 3,088 | |  | | 7.64 | | 7.57 | | 7.71 | | |  |  |  |
| **Know family planning source** | | | |  | |  | |  | |  | |  | | |  |  |  |
| No | | | | 2,270 | |  | | 7.27 | | 7.16 | | 7.38 | | |  |  |  |
| Yes | | | | 2,598 | |  | | 7.52 | | 7.43 | | 7.62 | | |  |  |  |
| **Ever had stillbirth/miscarriage** | | | |  | |  | |  | |  | |  | | |  |  |  |
| No | | | | 3,853 | |  | | 7.41 | | 7.32 | | 7.50 | | |  |  |  |
| Yes | | | | 1,015 | |  | | 7.40 | | 7.28 | | 7.53 | | |  |  |  |
| **Sibling had a maternal death** | | | |  | |  | |  | |  | |  | | |  |  |  |
| No | | | | 4,783 | |  | | 7.40 | | 7.32 | | 7.49 | | |  |  |  |
| Yes | | | | 85 | |  | | 7.57 | | 7.25 | | 7.89 | | |  |  |  |
| **Pregnancy complication** | | | |  | |  | |  | |  | |  | | |  |  |  |
| No | | | | 3,818 | |  | | 7.38 | | 7.29 | | 7.47 | | |  |  |  |
| Yes | | | | 1,050 | |  | | 7.51 | | 7.39 | | 7.63 | | |  |  |  |
| **Serious pregnancy complication** | | | | | |  | |  | |  | |  | | |  |  |  |
| No | | | | 3,996 | |  | | 7.36 | | 7.27 | | 7.45 | | |  |  |  |
| Yes | | | | 872 | |  | | 7.64 | | 7.53 | | 7.76 | | |  |  |  |
