# Supplementary material for: Rural/Urban and Socioeconomic Differentials in Quality of Antenatal Care in Ghana
Source: PLoS One. 2015 Feb 19;10(2):e0117996. doi: 10.1371/journal.pone.0117996 (PMC4335004; doi:10.1371/journal.pone.0117996)
Supplement: S3 Table — (DOCX) [file pone.0117996.s004.docx]

| **Table S3: Cross tabulation of key study variables by place of residence, education and wealth, GMHS, N = 5,042** | | | | | | | | | | | | | |
| --- | --- | --- | --- | --- | --- | --- | --- | --- | --- | --- | --- | --- | --- |
|  | *Total* |  | *Proportion in a rural area* | | |  | *Mean years of schooling* | | |  | *Proportion in richer/richest wealth tertile* | | |
| *Variable* | N |  | Proportion | [95% CI] | |  | Mean | [95% CI | |  | Proportion | [95% CI | |
| **Setting** |  |  |  |  |  |  |  |  |  |  |  |  |  |
| Rural | 3,115 |  |  |  |  |  | 4.019 | 3.657 | 4.381 |  | 0.157 | 0.124 | 0.189 |
| Urban | 1,927 |  |  |  |  |  | 6.938 | 6.535 | 7.341 |  | 0.776 | 0.726 | 0.827 |
| **Highest Education** |  |  |  |  |  |  |  |  |  |  |  |  |  |
| None | 1,697 |  | 0.822 | 0.787 | 0.857 |  | 0.000 |  |  |  | 0.159 | 0.125 | 0.193 |
| Primary | 1,109 |  | 0.689 | 0.648 | 0.731 |  | 4.187 | 4.077 | 4.297 |  | 0.347 | 0.304 | 0.390 |
| Middle/JSS | 1,830 |  | 0.559 | 0.509 | 0.608 |  | 8.726 | 8.673 | 8.780 |  | 0.491 | 0.445 | 0.537 |
| Secondary/SSS/higher | 406 |  | 0.276 | 0.214 | 0.339 |  | 12.458 | 12.214 | 12.702 |  | 0.814 | 0.758 | 0.869 |
| **Household wealth index** |  |  |  |  |  |  |  |  |  |  |  |  |  |
| Poorest | 1,097 |  | 0.969 | 0.946 | 0.992 |  | 2.358 | 1.982 | 2.735 |  |  |  |  |
| Poorer | 994 |  | 0.914 | 0.879 | 0.948 |  | 3.747 | 3.271 | 4.222 |  |  |  |  |
| Middle | 951 |  | 0.744 | 0.694 | 0.795 |  | 5.136 | 4.703 | 5.570 |  |  |  |  |
| Richer | 995 |  | 0.406 | 0.344 | 0.467 |  | 6.278 | 5.909 | 6.648 |  |  |  |  |
| Richest | 1,005 |  | 0.132 | 0.093 | 0.171 |  | 8.355 | 7.988 | 8.722 |  |  |  |  |
| **Region** |  |  |  |  |  |  |  |  |  |  |  |  |  |
| Greater Accra | 636 |  | 0.226 | 0.156 | 0.296 |  | 6.850 | 6.121 | 7.579 |  | 0.801 | 0.724 | 0.878 |
| Central | 441 |  | 0.637 | 0.557 | 0.717 |  | 5.837 | 5.122 | 6.552 |  | 0.380 | 0.268 | 0.492 |
| Western | 382 |  | 0.716 | 0.625 | 0.807 |  | 5.777 | 4.884 | 6.671 |  | 0.458 | 0.337 | 0.580 |
| Volta | 407 |  | 0.722 | 0.557 | 0.886 |  | 5.107 | 3.748 | 6.466 |  | 0.318 | 0.158 | 0.477 |
| Eastern | 744 |  | 0.661 | 0.604 | 0.719 |  | 6.238 | 5.757 | 6.720 |  | 0.358 | 0.286 | 0.431 |
| Ashanti | 855 |  | 0.620 | 0.552 | 0.688 |  | 6.451 | 5.873 | 7.028 |  | 0.473 | 0.405 | 0.541 |
| Brong Ahafo | 496 |  | 0.700 | 0.632 | 0.767 |  | 4.832 | 3.966 | 5.699 |  | 0.222 | 0.151 | 0.293 |
| Northern | 541 |  | 0.803 | 0.733 | 0.873 |  | 1.661 | 0.934 | 2.389 |  | 0.152 | 0.063 | 0.241 |
| Upper East | 303 |  | 0.790 | 0.716 | 0.865 |  | 1.648 | 0.803 | 2.494 |  | 0.189 | 0.099 | 0.280 |
| Upper West | 237 |  | 0.897 | 0.843 | 0.950 |  | 1.943 | 0.825 | 3.061 |  | 0.119 | 0.057 | 0.181 |
|  |  |  |  |  |  |  |  |  |  |  |  |  |  |
| **ANC quality of care score** |  |  |  |  |  |  |  |  |  |  |  |  |  |
| 7 or less | 1,901 |  | 0.737 | 0.699 | 0.775 |  | 4.074 | 3.709 | 4.439 |  | 0.278 | 0.236 | 0.320 |
| 8 or 9 | 2,967 |  | 0.591 | 0.554 | 0.627 |  | 5.808 | 5.490 | 6.127 |  | 0.443 | 0.405 | 0.481 |
| **No. of ANC visits** |  |  |  |  |  |  |  |  |  |  |  |  |  |
| 1-3 visits | 990 |  | 0.842 | 0.812 | 0.871 |  | 3.622 | 3.249 | 3.994 |  | 0.157 | 0.124 | 0.191 |
| Four or more | 3,878 |  | 0.599 | 0.564 | 0.634 |  | 5.512 | 5.181 | 5.843 |  | 0.435 | 0.396 | 0.473 |
| **Trimester of first ANC visit** |  |  |  |  |  |  |  |  |  |  |  |  |  |
| First trimester | 2,688 |  | 0.615 | 0.576 | 0.653 |  | 5.452 | 5.107 | 5.797 |  | 0.439 | 0.396 | 0.481 |
| Second trimester | 1,992 |  | 0.683 | 0.650 | 0.716 |  | 4.854 | 4.513 | 5.196 |  | 0.313 | 0.279 | 0.346 |
| Third trimester | 181 |  | 0.759 | 0.689 | 0.829 |  | 3.554 | 2.867 | 4.241 |  | 0.228 | 0.161 | 0.296 |
| Don't know | 7 |  | 0.647 | 0.227 | 1.067 |  | 1.434 | -0.346 | 3.214 |  | 0.285 | -0.129 | 0.699 |
| **Table S3 continued** |  |  |  |  |  |  |  |  |  |  |  |  |  |
| **Where ANC took place** |  |  |  |  |  |  |  |  |  |  |  |  |  |
| Gov't Health facility only or combine | 4,119 |  | 0.666 | 0.632 | 0.701 |  | 4.992 | 4.659 | 5.326 |  | 0.358 | 0.321 | 0.395 |
| Gov't hospital or polyclinic | 2,200 |  | 0.517 | 0.466 | 0.568 |  | 6.089 | 5.697 | 6.482 |  | 0.519 | 0.470 | 0.567 |
| Other Gov't facility | 1,919 |  | 0.835 | 0.789 | 0.880 |  | 3.750 | 3.327 | 4.174 |  | 0.177 | 0.142 | 0.212 |
| Only Private facility/maternity home | 703 |  | 0.535 | 0.448 | 0.623 |  | 6.058 | 5.328 | 6.788 |  | 0.507 | 0.424 | 0.589 |
| Home/other/DK | 46 |  | 0.699 | 0.533 | 0.865 |  | 3.357 | 2.125 | 4.590 |  | 0.279 | 0.121 | 0.436 |
| **Highest trained ANC provider** | |  |  |  |  |  |  |  |  |  |  |  |  |
| Doctor | 1,006 |  | 0.427 | 0.374 | 0.479 |  | 6.798 | 6.355 | 7.241 |  | 0.629 | 0.580 | 0.677 |
| Nurse | 3,743 |  | 0.697 | 0.664 | 0.730 |  | 4.764 | 4.437 | 5.091 |  | 0.321 | 0.284 | 0.357 |
| All others | 119 |  | 0.870 | 0.793 | 0.947 |  | 3.307 | 2.168 | 4.446 |  | 0.226 | 0.103 | 0.349 |
| **Reason for seeking ANC** |  |  |  |  |  |  |  |  |  |  |  |  |  |
| For checkup | 4,044 |  | 0.640 | 0.607 | 0.674 |  | 5.072 | 4.742 | 5.401 |  | 0.380 | 0.343 | 0.418 |
| For a problem/9missing | 824 |  | 0.685 | 0.644 | 0.726 |  | 5.415 | 4.977 | 5.853 |  | 0.370 | 0.321 | 0.419 |
